# Ranibizumab versus laser therapy for the treatment of very low birthweight infants with retinopathy of prematurity (RAINBOW): five-year outcomes of a randomised trial

**DOI:** 10.1016/j.eclinm.2024.102567

**Published:** 2024-04-11

**Authors:** Neil Marlow, James D. Reynolds, Domenico Lepore, Alistair R. Fielder, Andreas Stahl, Han Hao, Annemarie Weisberger, Amit Lodha, Brian W. Fleck

**Affiliations:** a UCL Elizabeth Garrett Anderson Institute for Women's Health, University College London, London, UK; b Ross Eye Institute, Jacobs School of Medicine and Biomedical Sciences, University at Buffalo, Buffalo, USA; c Department of Neuroscience, Sensory Organs and Thorax, Catholic University of the Sacred Heart, Gemelli Foundation IRCSS, Rome, Italy; d Department of Optometry & Visual Science, City, University of London, UK; e Department of Ophthalmology, University Medical Center Greifswald, Germany; f China Novartis Institutes for BioMedical Research Company Ltd; g Novartis Pharma AG, Basel, Switzerland; h Novartis Pharmaceuticals Corporation, East Hanover, NJ, USA; i Centre for Clinical Brain Sciences, University of Edinburgh, UK

**Keywords:** Infant, Preterm, Retinopathy of prematurity, Anti-VEGF treatment, Randomised controlled trial, Neurodevelopment

## Abstract

**Background:**

Concerns remain over the long-term safety of vascular endothelial growth factor (VEGF) inhibitors to treat retinopathy of prematurity (ROP). RAINBOW is an open label randomised trial comparing intravitreal ranibizumab (in 0.2 mg and 0.1 mg doses) with laser therapy in very low birthweight infants (<1500 g) with ROP.

**Methods:**

Of 201 infants completing RAINBOW, 180 were enrolled in the RAINBOW Extension Study. At 5 years, children underwent ophthalmic, development and health assessments. The primary outcome was visual acuity in the better-seeing eye. The study is registered with ClinicalTrial.gov, NCT02640664.

**Findings:**

Between 16-6-2016 and 21-4-2022, 156 children (87%) were evaluated at 5 years. Of 32 children with no acuity test result, 25 had a preferential looking test, for 4 children investigators reported low vision for each eye, and in 3 further children no vision measurement was obtained. 124 children completed the acuity assessment, the least square mean (95% CI) letter score in the better seeing eye was similar in the three trial arms—66.8 (62.9–70.7) following ranibizumab 0.2 mg, 64.6 (60.6–68.5) following ranibizumab 0.1 mg and 62.1 (57.8–66.4) following laser therapy; differences in means: ranibizumab 0.2 mg v laser: 4.7 (95% CI: −1.1, 10.5); 0.1 mg v laser: 2.5 (−3.4, 8.3); 0.2 mg v 0.1 mg: 2.2 (−3.3, 7.8). High myopia (worse than −5 dioptres) in at least one eye occurred in 4/52 (8%) children following ranibizumab 0.2 mg, 8/55 (15%) following ranibizumab 0.1 mg and 11/45 (24%) following laser therapy (0.2 mg versus laser: odds ratio: 3.99 (1.16–13.72)). Ocular and systemic secondary outcomes and adverse events were distributed similarly in each trial arm.

**Interpretation:**

5-year outcomes confirm the findings of the original RAINBOW trial and a planned interim analysis at 2 years, including a reduced frequency of high myopia following ranibizumab treatment. No effects of treatment on non-ocular outcomes were detected.

**Funding:**

10.13039/100008792Novartis Pharma AG.

**Research in context**

**Evidence before this study**

Intravitreal anti-vascular endothelial growth factor (VEGF) agents are used to treat retinopathy of prematurity. We searched for “retinopathy of prematurity” AND “anti-VEGF” in 6 databases from 1st January 2021 to 17 October 2023 (from publication of our 2-year interim analysis to current). A meta-analysis (2 RCTs and 11 retrospective studies) of refractive status at a variety of ages showed rates of myopia were lower following anti-VEGF, compared to laser, but two recent retrospective studies of refractive status at age 4–6 years found no differences. One small retrospective study found no difference in visual acuity following bevacizumab compared to laser but a second reported reduced visual acuity in eyes treated with ranibizumab compared to untreated eyes with mild ROP.

**Added value of this study**

Our results for visual acuity at 5 years demonstrate that ranibizumab 0.2 mg results in visual acuity at least as good as that obtained following laser.

**Implications of all the available evidence**

These results provide evidence that ranibizumab is a safe and effective treatment for ROP with comparable results to laser treatment.

## Introduction

Despite the increasing use of intravitreal anti-vascular endothelial growth factor (VEGF) agents to treat retinopathy of prematurity (ROP),^1^, ^2^, ^3^, ^4^ there are relatively few data on infant outcomes from controlled trials^5^ and no data describing outcomes into school age. Such data are important as there remains concern that the suppression of systemic VEGF levels after intravitreal treatment might produce remote effects on somatic or neurological development.^6^ For example, recent reports,^7^, ^8^, ^9^ including two systematic reviews with meta-analysis,^10^^,^^11^ have suggested that neuromorbidity may be more frequent after bevacizumab treatment. The two largest non-randomised studies^7^^,^^8^ comprise the majority of included infants and may have been biased because anti-VEGF treatment was preferred in sicker and less mature infants, who would be expected to have higher rates of neurodevelopmental impairment. There remains a need to demonstrate long term safety following anti-VEGF therapy.

Furthermore, there are limited data on long term refractive status, ocular structures and visual functions. One systematic review published in 2022 concluded that more information is required concerning the adverse events, complications, and unfavourable functional and structural outcomes of the two treatment modalities in the long term.^12^ In the interim analysis at 2 years of 180 infants in the RAINBOW trial,^13^ as among 109 children at 2.5 years in the BEAT-ROP trial,^14^ there was significant reduction of high myopia following anti-VEGF treatment compared to laser, but no visual acuity data have been published following a trial of anti-VEGF therapy for ROP.

In the ranibizumab versus laser therapy for the treatment of very low birthweight infants with retinopathy of prematurity trial (RAINBOW) we have demonstrated that ranibizumab 0.2 mg (ranibizumab) might be superior to laser treatment of retinopathy, with fewer unfavourable ocular outcomes and an acceptable safety profile 24 weeks following treatment.^15^ In an interim analysis of the five year RAINBOW Extension Study, which described the status of infants at 2 years of age,^13^ there were no new ocular findings and, as reported above, high myopia (worse than −5 dioptres) was less common after ranibizumab 0.2 mg compared to laser therapy. Importantly, somatic and developmental outcomes did not differ significantly between groups and there was possibly better vision related quality of life after ranibizumab. Status at 2 years may be too early to determine precise ocular findings and to detect safety issues and a longer period of follow up is recommended.^16^

We now report the per protocol outcome for children enrolled into the RAINBOW Extension Study through to 5 years of age to ascertain visual acuity, the most important functional outcome. Our aim was to determine if the benefits of ranibizumab persist, and whether any new safety findings have arisen.

## Methods

### Study design and participants

**Fig. 1:**
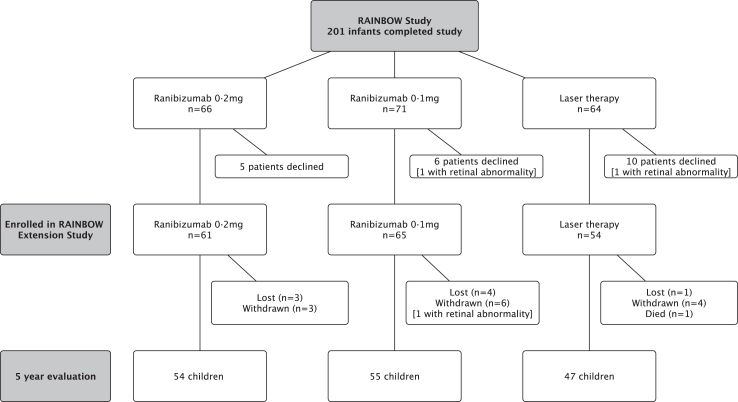
Consort diagram for children who completed RAINBOW and entered the RAINBOW extension study.

RAINBOW was an open-label, randomised controlled trial of ranibizumab 0.2 mg or 0.1 mg compared with laser therapy (1:1:1) in very low birth weight infants (<1500 g) with a diagnosis of bilateral ROP Zone I stage 1+, 2+, 3 or 3+, or Zone II stage 3+, or aggressive posterior ROP. Stratification was by baseline ROP Zone and geographical region.^15^ The trial recruited in 87 centres in 26 countries. The RAINBOW core study reported a composite primary outcome of disease-free survival without structural retinal abnormalities at 24 weeks or the need to switch to a different treatment modality. At the completion of the study 201 infants were eligible to enter the RAINBOW extension study and the parents of 180 children consented to take part (Fig. 1).

### Ethics

The study was approved by the independent ethics committee or institutional review board of each study centre and was conducted in accordance with the Declaration of Helsinki and International Conference on Harmonization Good Clinical Practice Guidelines.^17^ Parents or guardians provided written informed consent to participation in this study.

### Procedures

A comprehensive ophthalmic assessment was performed at age 5 years, ± 12 weeks by trained study ophthalmologists.

**Visual acuity** was assessed by a certified examiner, masked to treatment allocation. Lea symbols in a LogMAR Early Treatment of Diabetic Retinopathy Study (ETDRS) style array^18^ were presented using a light box at 3 m (1 m if less than 20 symbols were identified at 3 m). The child's current prescription glasses or contact lenses were worn. If initial visual acuity measurement was less than 75 symbols (LogMAR 0.2; Snellen 20/32), a non-mydriatic refraction was performed and optical correction optimised. Testing was uniocular, results expressed as the number of symbols correctly identified. For children with apparent cognitive or developmental impairment who were unable to perform acuity measurement, a binocular pre-verbal Cardiff Acuity Card (CAC) preferential looking test^19^ at 1 m was performed. CAC optotypes consist of pictures drawn with a white line bordered by two black lines. If optotype line separation falls below the subject's acuity limit, the optotype merges with the grey background. The narrowest line separation giving a visible optotype to which the child responds is read as their visual acuity, reported in LogMAR units. If visual acuity was so poor that ETDRS or CAC testing could not be performed, low vision “counting fingers”, “hand movements” and “light perception” tests were performed.

**Uniocular central fixation behaviour and “confrontation” peripheral visual field extent** were then assessed. Nystagmus or strabismus in the primary position was noted. Direct and consensual pupil reactions were examined using a flashlight. After cycloplegia, **refraction** was performed using retinoscopy or auto-refraction. **Fundus examination** was performed using indirect ophthalmoscopy. The circumferential extent of **full peripheral retinal vascularization** by clock hour was recorded.

Parents completed the **Children's Visual Function Questionnaire** (CVFQ)^20^ version for children aged more than 3 years. This validated Likert scale questionnaire comprises four vision-related subscales (competence, personality, family impact, and treatment effect), general health and general vision subscales, and a summative composite score. Subscale and summary scores are standardised to range from 0 to 100. Completed questionnaires were reviewed by parents and research staff at a face-to-face meeting during the 5-year evaluation.

**Non-ocular outcomes** were assessed by trained local study teams. Developmental progress was assessed using the Mullen Scales of Early Learning^21^ (visual reception, and receptive and expressive language), and motor function assessed according to the Gross Motor Function Classification System.^22^^,^^23^ All assessors underwent web-based training and following a post-training knowledge quiz, were considered certified. A detailed training manual for administration of all evaluations, specifically including the Mullen Scales of Early Learning, was provided to each site and assessments were made in the most familiar language of the family. Growth data (standing height and weight) were collected and converted to Z scores based on age corrected for weeks of prematurity to maintain parity with 2-year data. Participants’ most recent blood pressure values, duration of rehospitalisation, and use of concomitant medications were obtained from clinical records. Respiratory morbidity and hearing function were determined from parent questionnaire completed at the 5-year assessment visit.

Investigator-determined ocular and non-ocular serious and other adverse events as defined in the protocol were recorded in clinical record forms at each follow-up visit and uploaded to the study database. Here we report adverse events from enrolment in the RAINBOW core study up to 5 years of age. Frequencies of refractive errors and ocular outcomes such as strabismus, nystagmus, abnormal fixation, and abnormal pupil reaction are based on ophthalmic examination results, although some investigators additionally reported such outcomes as adverse events.

### Statistics

Infants eligible for the RAINBOW study were randomized in a 1:1:1 ratio to receive bilateral intravitreal ranibizumab 0.2 mg, intravitreal ranibizumab 0.1 mg or laser therapy at baseline (day 1, treatment phase), using interactive response technology, stratified by the ROP zone of the worse eye and geographic region.^15^ The core trial was powered for the primary composite efficacy outcome determined up to 24 weeks after starting investigational treatment. At this point 201 infants became eligible to join the RAINBOW extension study (Fig. 1). Thus, the extension study was not powered nor randomised for 5-year outcome; the results reported may thus be considered descriptive.

### Outcome measures

The primary outcome for the 5-year analysis was visual acuity in the better-seeing eye at age 5 years. Secondary outcomes were the other results of the 5-year ophthalmic assessment, refractive error, vision related quality of life and non-ocular outcomes, as described above.

Selected key comparisons between the ranibizumab 0.2 mg, ranibizumab 0.1 mg, and laser therapy groups were done, using tests defined in the core study. The primary outcome, visual acuity at age 5 years, was assessed using analysis of covariance (ANCOVA), with adjustment for baseline ROP zone which we considered the primary confounder. Categorical data, such as proportions of children with high myopia or with abnormal retinal structure were analysed using Cochran-Mantel-Haenszel (CMH) test, with baseline ROP Zone as a factor. Mullen Scales of Early Learning and growth parameters were compared using the Z test for odds ratios (OR) and analysis of covariance (ANCOVA) was used to compare continuous data with and without adjustment for gestational age and geographical region; gestational age is closely associated with cognitive outcome and region was included an important confounder. 95% confidence intervals were used to report significance at the 5% level. All statistical analyses were done using SAS, version 9.4. This trial is registered with ClinicalTrials. gov, NCT02640664.

### Role of the funding source

The funder of the study had full access to and was involved in data collection, data analysis, and data interpretation, and was involved in the writing of the manuscript and the decision to submit.

## Results

The RAINBOW core study^15^ reported outcomes at 24 weeks following trial interventions based upon the composite of survival without structural eye abnormalities or the need to switch to a different treatment modality; 180/201 surviving infants were enrolled in the RAINBOW Extension Study; between 16-6-2016 and 21-4-2022 (87% of those enrolled) completed the 5-year evaluation (Fig. 1). There were two deaths in the laser arm unrelated to the trial intervention 9 and 22 months after enrolment, respectively, reported previously.^13^ The distribution of baseline characteristics at enrolment into RAINBOW were similar for those evaluated at 5 years and those in the core study (Table 1).

**Table 1:** Comparison of baseline characteristics for those evaluated at 5 years in the RAINBOW extension study and the core study population from RAINBOW.

Entered into extension study	Ranibizumab 0.2 mg	Ranibizumab 0.1 mg	Laser	Core study population
	61	65	54	n = 225
Gestational age at birth				
Median [range]	25.0 [23–32] weeks	26.0 [23–32] weeks	26.0 [23–32] weeks	26 [23–32] weeks
No. ≤ 24 weeks	25 (41%)	21 (32%)	19 (35%)	37%
>24–<27 weeks	16 (26%)	19 (29%)	11 (20%)	25%
≥27 weeks	20 (33%)	25 (38%)	24 (44%)	38%
Male Sex	29 (48%)	30 (46%)	27 (50%)	107 (48%)
Multiple birth	16/58 (28%)	16/63 (25%)	16/49 (33%)	54/207 (26%)
Geographical region				
Region 1 (NNM^a^ ≤5 × 10^3^)	38 (62%)	41 (63%)	33 (61%)	134 (60%)
Region 2 (NNM^a^ >5 × 10^3^)	23 (38%)	24 (37%)	21 (39%)	91 (40%)
Ethnic group				
White	38 (62%)	40 (62%)	32 (59%)	110 (61%)
Black	0	4 (6%)	2 (4%)	6 (3%)
Asian	22 (36%)	18 (28%)	18 (33%)	58 (32%)
Other	1 (2%)	3 (5%)	2 (4%)	6 (3%)
Postmenstrual age core study entry (median [range])	37.0 [30.3–51.9] weeks	36.9 [31.9–44.6] weeks	36.3 [31–47.6] weeks	
Postmenstrual age at extension study entry (w)	63.1 [56.7–95.1] weeks	64.0 [56.6–106.1] weeks	61.7 [55–99.6] weeks	
Site of ROP at core study enrolment^b^				
Zone I	23 (38%)	25 (38%)	21 (39%)	86 (38%)
Zone II	38 (62%)	40 (62%)	33 (61%)	138 (62%)^b^
Aggressive posterior-ROP at core study enrolment	7 (11%)	8 (12%)	8 (15%)	30 (13%)
Worst grade of ROP/subject at core study enrolment				
Zone I AP ROP	7	8	7	29
Zone II AP ROP	–	–	1	1
Zone I stage 3+	10	11	8	37
Zone I stage 3	3	4	1	8
Zone I stage 2+	3	1	4	9
Zone I stage 1+	–	1	1	3
Zone II stage 3+	38	39	32	135
Zone II stage 3	–	1	–	1
Zone II stage 2+				1
Completed 5 year study	54 (89%)	55 (85%)	47 (87%)	

a NNM, neonatal mortality in respective countries at time of core study.

b 1 patient (who received ranibizumab 0.1 mg) had no Zone specified.

### Primary outcome

**Fig. 2:**
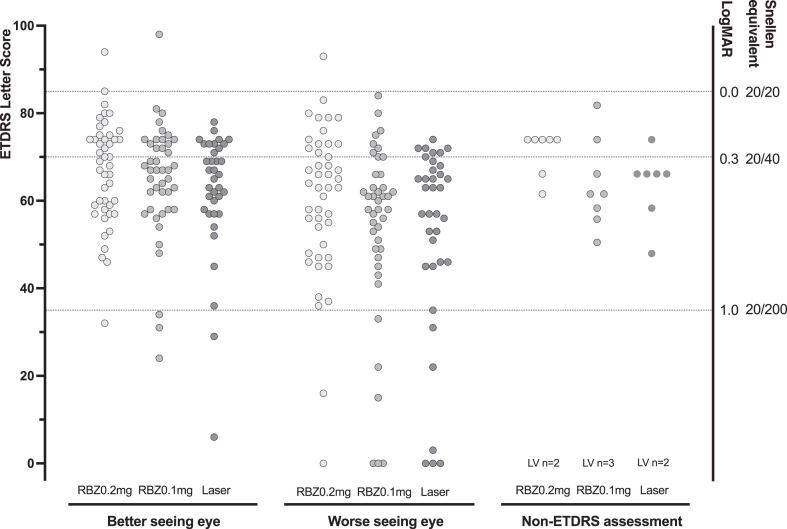
Visual acuity for children evaluated at 5 years in the RAINBOW Extension Study. Individual results are shown as dots in study groups as ranibizumab 0.2 mg (RBZ0.2 mg: light grey), ranibizumab 0.1 mg (RBZ0.1 mg: mid grey), and laser (dark grey). Results are shown for the better and worse seeing eyes, and separately for children who received only a Cardiff Card binocular assessment; LV indicates a small number of children were recorded as having low or no vision without Early Treatment Diabetic Retinopathy Study (ETDRS) chart Letter Score results or Cardiff Acuity Card results. In three further children (not shown) no vision could be measured. Y axes show ETDRS Letter score **(left y axis)**, equivalent LogMAR value or Snellen chart equivalent (right y axis and dotted lines).

ETDRS visual acuity was assessed at 5 years in 124/156 (79%) participants (Table 2). Among the 124 children tested with ETDRS, the Least Square (LS) mean (95% CI) letter score in the better-seeing eye was similar in the three groups: 66.8 (62.9–70.7) for ranibizumab 0.2 mg, 64.6 (60.6–68.5) for ranibizumab 0.1 mg and 62.1 (57.8–66.4) for laser therapy (Table 2; Fig. 2). A letter score of 65 letters is equivalent to a Snellen acuity of 20/50. Differences in means were ranibizumab 0.2 mg v laser: 4.7 (95% CI: −1.1, 10.5); 0.1 mg v laser: 2.5 (−3.4, 8.3); 0.2 mg v 0.1 mg: 2.2 (−3.3, 7.8). Of 32 children who completed follow up to 5 years without an ETDRS result, Cardiff Acuity Card (CAC) results were obtained in 25 children. For 4 further children investigators reported low vision for each eye and in 3 children no vision measurement was obtained, one in each trial group. CAC and Low Vision results were also similar in the three treatment groups (Fig. 2). When CAC or LV results were added, 2 children in the ranibizumab 0.2 mg group, 5 children in the ranibizumab 0.1 mg group and 3 children in the laser group had a visual acuity equivalent to less than 20/200 in the better-seeing eye and may be considered severely vision impaired.

**Table 2:** Ocular outcomes for children evaluated at 5 years in the RAINBOW extension study.

Entered into extension study	Ranibizumab 0.2 mg	Ranibizumab 0.1 mg	Laser
	n = 61	n = 65	n = 54
**Primary outcome**			
Visual Acuity (evaluated per eye)			
ETDRS assessed at 5 y	45/54 (83%)	43/55 (78%)	36/37 (77%)
Acuity results			
Better seeing eye (letter score) LS mean (SE)	66.8 (1.95) (n = 45)^a^	64.6 (2.00) (n = 43)^a^	62.1 (2.18) (n = 36)^a^
Worse seeing eye (letter score) LS mean (SE)	60.2 (2.95) (n = 45)	53.8 (3.05) (n = 42)	52.2 (3.30) (n = 36)
CAC Binocular (logMar) median (range), n	0.1 (0.1–0.3) (n = 7)	0.2 (−0.1 to 0.8) (n = 9)	0.2 (−0.1 to 1.0) (n = 9)
Visual acuity 70 letters (Snellen 20/40) or better in the better-seeing eye	20/45 (44%)	15/43 (35%)	11/36 (31%)
Severe visual impairment (<20/200) in better-seeing eye	2	5	3
**Secondary outcomes**			
Structural abnormality macula	1/60 (2%)	4/65 (6%)	6/53 (11%)
Normal macula, pre-retinal fibrosis present^b^	1/60 (2%)	3/65 (5%)	2/53 (4%)
Full vascularisation at 5 years	30/53 (57%)	23/56 (42%)	n/a
Visual fields minor abnormality	2	2	6
Major abnormality			1
Refraction at 5 years: mean (SD) spherical equivalent (dioptres) (n eyes)	−0.75 (2.82) (n = 104)	−0.97 (2.88) (n = 104)	−1.79 (4.04) (n = 90)
*(Equivalent data from 2 year assessment) (n eyes)*	*−0.73 (2.70) (n* = *110)*	*−0.89 (2.79) (n* = *98)*	*−1.58 (4.07) (n* = *82)*
High myopia (−5 dioptres or worse in either eye) per child	4/52 (8%)^c^	8/55 (15%)	11/45 (24%)
Refraction excluding eyes that received any treatment with laser (n eyes)	−0.60 (2.70) (n = 97)	−0.85 (2.83) (n = 98)	–
**Ocular functions assessed at 5 years**	**52 (104 eyes)**	**55 (110 eyes)**	**47 (94 eyes)**
Nystagmus	1 (2%)	5 (9%)	2 (4%)
Strabismus	14 (17%)	15 (27%)	17 (33%)
Esotropia	7 (13%)	8 (15%)	11 (23%)
Exotropia	5 (10%)	6 (12%)	6 (12%)
Abnormal fixation, children (eyes)	1 (1 eye)	4 (5 eyes)	4 (5 eyes)
Abnormal pupil, children (eyes)	0	0	1 (1 eye)

a Difference in LS means (95% confidence interval): ranibizumab 0.2 mg v laser: 4.7 (−1.1 to 10.5); ranibizumab 0.1 mg v laser: 2.5 (−3.4 to 8.3); ranibizumab 0.2 mg v 0.1 mg: 2.2 (−3.3 to 7.8), based on a stratified analysis of variance (ANOVA) model with VA as the continuous response variable, ROP zone (I and II) at core baseline and treatment arm as factors.

b In addition, one child had pre-retinal fibrosis associated with stage 4 A retinal detachment (normal macula) and one child had pigment disturbance at the macula.

c Odds Ratio (95% confidence interval) ranibizumab 0.2 mg v laser: 3.99 (1.16–13.72).

### Secondary ophthalmic outcomes

No further child developed structural abnormalities after the core study report at 24 weeks, and, apart from one infant in the ranibizumab 0.1 mg group given a second reinjection on the first day of the extension study, no other treatments for ROP were given during the extension study.

At 5 years, ocular outcomes were reported for 156/180 children enrolled in the extension study (Table 2). Structural abnormality involving the macula, was reported in 1 child following ranibizumab 0.2 mg, 4 following ranibizumab 0.1 mg and 6 following laser; ranibizumab 0.2 mg versus laser OR (95% CI) 7.77 (0.88–68); ranibizumab 0.1 mg versus laser: 1.95 (0.52–7.36); ranibizumab 0.2 mg v 0.1 mg: 3.92 (0.42–37). Less severe abnormalities were also reported and were least frequent in the ranibizumab 0.2 mg group (Table 2).

Refraction was performed in 303 eyes of 153 children (Supplemental Figure S1). The mean (SD) spherical equivalent was −0.75 (2.82) in 104 eyes in the ranibizumab 0.2 mg group, −0.97 (2.88) in 109 eyes in the ranibizumab 0.1 mg group and −1.79 (4.04) in 90 eyes in the laser group. Equivalent data from the 2-year analysis is shown in Table 2 for comparison. After excluding eyes that had also received laser, results in the ranibizumab groups were slightly less myopic, the mean was −0.60 (2.70) in 97 eyes in the ranibizumab 0.2 mg group and −0.85 (2.83) in 98 eyes in the ranibizumab 0.1 mg group (Table 2). Myopia of −5.00 dioptres or worse was present in 6/104 (6%) eyes in the ranibizumab 0.2 mg group, 10/109 (9%) in the ranibizumab 0.1 mg group and 17/90 (19%) in the laser group. Myopia of −5.00 dioptres or worse was present in at least one eye of 4/52 (8%) children in the ranibizumab 0.2 mg group, 8/55 (15%) in the ranibizumab 0.1 mg group and 11/45 (24%) in the laser group. 0.2 mg versus laser OR (CI) 3.99 (1.16–13.72).

The frequency of strabismus, nystagmus, abnormal fixation, or abnormal pupil reaction was similar between treatment groups (Table 2). Full peripheral retinal vascularisation in both eyes was identified in 30/53 (57%) infants treated with ranibizumab 0.2 mg and 23/56 (42%) infants treated with ranibizumab 0.1 mg. Clinical assessment of children's binocular visual field indicated that two children in each of the ranibizumab groups had “minor impairment”, in the laser group six children had “minor impairment” and one child “major impairment”.

Between enrolment in the core study through 5 years, new adverse ocular events were reported in 18 children following ranibizumab 0.2 mg, 25 children following ranibizumab 0.1 mg, and 22 children following laser therapy (Supplemental Table S1). Serious adverse events continuing from the core study or arising during the extension study were not reported in the 0.2 mg ranibizumab group, compared with 6 children in the 0.1 mg ranibizumab group, and 2 in the laser group. Apart from one case of strabismus in the laser group, no adverse events were attributed to a study treatment by the investigators. The distributions of CVFQ scores were similar in each study group for composite, general or vision health, and other subscales (Supplemental Figure S2).

### Other outcomes

**Fig. 3:**
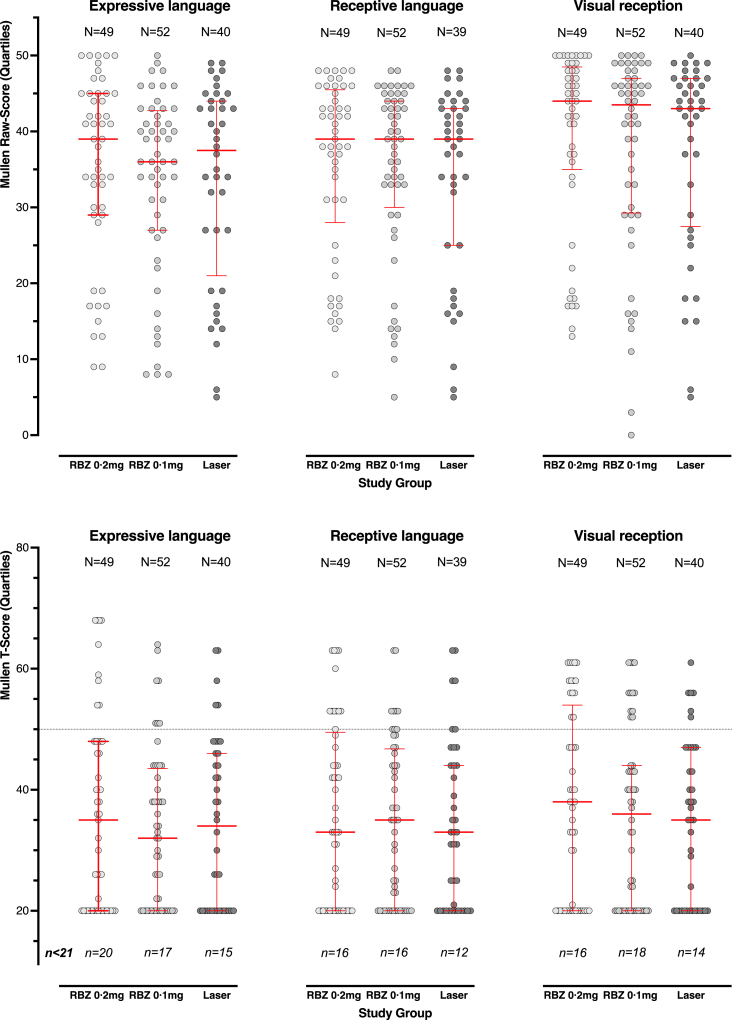
T-scores (top graphs) and Raw scores (bottom graphs) from the Mullen Scales of Early Learning at 5 years among children evaluated at 5 years in the RAINBOW extension study. The dotted line represents standardization population mean; error bars show the median with 25th and 75th percentiles.

Neurodevelopment at 5 years was evaluated using the Mullen Scales for 141/157 (90%) children. Using standardised T-scores (population mean 50), all study groups showed significant reduction in mean scores compared to the normative population, as anticipated in this population of very low birthweight children, but between study groups no significant differences were found in expressive language, receptive language, or visual recognition scores. However, in each group between 29 and 41% (mean 34%) scored below 20, the floor of the test. Examining individual raw scores, which are continuous, again revealed no differences (Fig. 3).

Gross motor function grades 2–5 indicating cerebral palsy with moderate or severe impairment were observed in 5 children after ranibizumab 0.2 mg, 5 children after ranibizumab 0.1 mg and 7 following laser, and moderate/severe hearing impairment in 1, 2, and 3 children in each group, respectively.

Height and weight were similarly distributed in all three groups (Table 3; Supplemental Figure S3) and there were no differences in systolic or diastolic blood pressure (Table 3). Small numbers of families reported wheezing, cough and regular medication use that did not differ statistically between groups. Systemic (non-ocular) serious AEs are listed in Supplemental Table S2 and similarly distributed between groups. Proportions of children with AEs related to the risk of neurodevelopmental impairment using pre-determined criteria were comparable between treatment arms; none were considered to be related to the study treatment/procedure by the Investigator (Supplemental Table S3).

**Table 3:** Neuroimpairment, somatic and respiratory outcomes for children evaluated at 5 years in the RAINBOW extension study.

	Ranibizumab 0.2 mg	Ranibizumab 0.1 mg	Laser
Entered into extension study	61	65	54
Completed 5 year evaluation	49	52	40
**Neurodisability:**			
Mullen scales (mean (sd) T-scores)			
Expressive language	37 (12)	35 (15)	35 (13)
Receptive language	36 (15)	35 (13)	34 (14)
Visual recognition	36 (16)	33 (13)	34 (14)
GMFCS			
Grade 2	2 (3%)	1 (2%)	5 (9%)
Grade 3	–	–	–
Grade 4–5	3 (5%)	4 (6%)	2 (4%)
Moderate/severe/profound hearing impairment	1 (2%)	2 (3%)	3 (5%)^a^
Severe visual impairment (<20/200)	2 (2%)	5 (9%)	3 (5%)
**Somatic outcomes:**			
Weight mean (sd) kg	16.5 (3.1)	16.3 (2.8)	16.6 (3.3)
Mean z score (sd)	−0.93 (1.5)	−1.0 (1.5)	−0.91 (1.5)
Height mean (sd) cm	106.7 (7.3)	105.8 (6.1)	106.5 (6.2)
Mean z score (sd)	−0.3 (1.5)	−0.43 (1.4)	−0.33 (1.3)
Systolic Blood Pressure	99.7 (13.7)	93.8 (10.6)	94.1 (10.8)
Diastolic blood pressure	62.9 (11.1)	58.5 (10.0)	60.0 (10.7)
Non-ocular adverse events	45 (75%)	53 (82%)	46 (85%)
**Respiratory outcomes**			
Wheezing (ever had)	6 (10%)	4 (6%)	2 (4%)
Number of episodes 1–3	2 (3%)	3 (5%)	1 (2%)
4–12	4 (5%)	–	1 (2%)
More than 12	–	1 (2%)	–
Frequency of sleep disturbance <1 night/week	5 (8%)	1 (2%)	–
1+ night/week	–	1 (2%)	–
Dry cough at night	5 (8%)	2 (3%)	1 (2%)
Regular medication for wheeze:	6 (10%)	9 (14%)	3 (6%)
Smoker at home	15 (25%)	8 (12%)	4 (7.4%)

GMFCS, Gross Motor Function Classification System for children with cerebral palsy (Grade 5 is most severe).

a One patient had profound hearing loss; all others were aided with improvement.

## Discussion

The RAINBOW core study to 24 weeks used a composite primary outcome of disease-free survival without structural abnormality of the retina or the need to switch to a different treatment modality and showed that ranibizumab 0.2 mg might be superior to laser therapy (P = 0.051).^15^ There were fewer unfavourable outcomes following ranibizumab 0.2 mg than laser therapy and no safety concerns. Interim analysis of the RAINBOW extension study at 2 years confirmed the core study ocular structure outcomes and in addition demonstrated reduced high myopia, possibly better vision-related quality of life, and no adverse non-ocular infant development.^13^ We now report the 5-year outcomes. ETDRS visual acuity in the better-seeing eye was similar in the three treatment groups, with the highest mean letter score observed in the ranibizumab 0.2 mg group. No new structural ocular abnormalities were reported. The prevalence of high myopia remained lower in the ranibizumab 0.2 mg group compared with laser therapy. The proportions of infants with strabismus, nystagmus, or abnormal ocular fixation were similar in the treatment groups and lowest following ranibizumab 0.2 mg. Parent-report of vision-related quality of life was similar in the three treatment groups. In terms of safety, developmental outcomes, respiratory health, blood pressure, and growth were similarly distributed between the three treatment groups and adverse events over the intervening 5 years were similarly distributed in each group. Ranibizumab 0.2 mg is efficacious and safe up to 5 years of age.

In the core study, there were higher odds of a successful outcome following ranibizumab 0.2 mg compared with 0.1 mg, although the differences were not statistically significant. At 5 years, the letter score visual acuity was higher in the ranibizumab 0.2 mg group, abnormal structure occurred less frequently, and the risk of high myopia was less (8% versus 15%). Other 5-year ophthalmic and developmental outcomes did not differ between the two ranibizumab groups. The dose of 0.2 mg ranibizumab has been licensed for the treatment of ROP in Europe.^24^ Worldwide, the only similar randomised trial, the FIREFLEYE trial, compared aflibercept 0.4 mg against laser (in a ratio of 2:1) in 118 infants, using a similar composite primary outcome to RAINBOW; at 24 weeks, treatment was successful in 86% infants in the aflibercept group and 82% infants in the laser group.^25^^,^^26^

Several publications call for safety assessment of anti-VEGF treatment into childhood.^6^^,^^27^ No new primary publications have reported beyond 2 years since we published our interim analysis. Several studies have evaluated developmental outcomes retrospectively in groups of children who received bevacizumab compared with either laser therapy or cryotherapy, with mixed outcomes. Two recent systematic reviews concluded that there was an association between bevacizumab and adverse neurodevelopmental outcomes, such that new safety studies were required including long term follow up.^10^^,^^11^ All anti-VEGF agents move quickly into the systemic circulation after intravitreal injection, but bevacizumab and aflibercept appear to be cleared more slowly from the circulation with resultant suppression of VEGF levels.^28^ We have confirmed the rapid clearance of ranibizumab and lack of effect on VEGF levels in the RAINBOW population.^29^ For ranibizumab, RAINBOW provides data giving confidence that the trend to benefit in terms of vision (better acuity and less high myopia) does not come at a systemic “cost”.

For the first-time we report visual acuity results in a treatment trial of anti-VEGF for ROP and thus the single most important functional outcome for affected patients. Letter scores were similar in the three treatment groups and were highest in the ranibizumab 0.2 mg group. Mean letter scores ranged from 62.1 to 66.8, equivalent to logMAR 0.458–0.364,^30^ or approximately Snellen 20/50, which is 5th percentile for a normal population of this age.^31^ Reduced acuity has been reported in an essentially normal-looking retina following spontaneous ROP regression^32^ and following its treatment by laser.^33^ This may be attributed to subtle foveal abnormalities/sequelae^34^^,^^35^ or to cerebral vision impairment,^36^ these two not being mutually exclusive, indeed the possibility of both being present in some children is high. Our data do not permit further speculation.

No new structural retinal abnormalities occurred in the extension study. This finding is reassuring not only for the management of acute disease, but also in relation to persistent avascular retina (PAR)^37^ following anti-VEGF treatment of ROP.^38^ The time to full retinal vascularisation is difficult to ascertain by clinical observation in young children, and our finding that 49% eyes treated with ranibizumab were fully vascularized at 5 years is likely an underestimate. Nevertheless, no infant in the RAINBOW extension study was treated for PAR or a complication of PAR.

Refractive errors, including hypermetropia, astigmatism, anisometropia, and myopia, are associated with both prematurity and ROP, particularly following stage 3 ROP or worse.^39^^,^^40^ In the untreated control eyes of the multicentre trial of cryotherapy for retinopathy of prematurity at 3.5 years of age, 27% of eyes had myopia of −6 D or worse.^41^ Although evaluated using different definitions and ages, high myopia is a frequent occurrence following severe ROP whether untreated or treated by cryotherapy or laser therapy.^41^^,^^42^ A recent meta-analysis^43^ showed less myopia following anti-VEGF treatment than following laser. In RAINBOW at 5 years, high myopia occurred more frequently in laser-treated infants occurring in 11 (24%) of 46 infants in the laser group compared to 4 (8%) of 52 infants in the ranibizumab 0.2 mg group. In the BEAT-ROP trial, ophthalmic follow-up at 2.5 years described a lower prevalence of very high myopia (defined as –8D or worse) following anti-VEGF treatment compared with laser therapy.^14^ In RAINBOW no progression of myopia occurred between 2 and 5 years, in keeping with results from the ETROP laser trial.^42^

RAINBOW was powered on eye outcomes at 24 weeks post-treatment leading to a lack of power to detect small differences in long term systemic outcomes. We have reported mainly descriptive outcomes that should be interpreted with caution. Furthermore, all follow-up studies have problems with attrition. We recruited into the extension study 90% of the population who completed the core study and were able to determine the outcome at 5 years for 156 infants (87% of those recruited into the extension study, or 78% of those completing the core study). Two patients died in the laser therapy group after completion of the core study from causes that appear unrelated to the trial but attrition was greatest in the laser group. There was no evidence of systematic bias in loss to follow-up among the evaluated population based on trial criteria; however, among preterm populations, social disadvantage, which was not recorded in our study, is generally known to be higher in those lost to follow-up and dropouts might comprise a greater proportion of children with disability. We used a robust developmental measure (Mullen Scales of Early Learning), chosen to provide an estimate of developmental attainment across the trial sites in the child's usual language. Despite the high prevalence of results below the standardised T-scores, using raw scores did not change the finding. We have identified no systemic safety concerns in cognition, respiratory symptoms, growth, or blood pressure to 5 years. It was not feasible to evaluate children for the presence of central visual impairment, which is well described in preterm populations due to limitations on available tools and difficulties in using questionnaires across different languages. Limitations to the ocular data include the difficulties of measuring visual acuity in 5-year-old ex-premature children. Uniocular ETDRS measurements were obtained in 79% of children who reached the 5-year assessment, and binocular CAC results were obtained in a further 16%. While visual acuity assessments were masked to treatment group, retinal examinations could not be masked to the presence of laser scars.

To conclude, in the RAINBOW Extension Study at 5 years of age corrected for prematurity, no effects of ranibizumab were detected on neurodevelopment, growth, blood pressure, or respiratory symptoms; and overall visual acuity, refractive status and retinal structure along with vision-related quality of life ratings were as least as good following ranibizumab 0.2 mg compared to laser. No late ocular complications affecting vision were seen. The results of anti-VEGF treatment seen in the RAINBOW trial have translated into a lower risk of high myopia at 5 years with no concerns about long-term safety.

## Contributors

The Study was conceived and designed by AS, DL, ARF, BWF, JDR, and NM in conjunction with Novartis Pharma AG. The Study was funded and fully managed by Novartis. AS, DL, ARF, BWF, JDR, and NM formed the Protocol Steering Committee. The statistical analysis plan was agreed a priori by all authors and undertaken by HH. All contributors had full access to the data. The first draft of the manuscript was written by NM and BWF, with equal contributions from DL, ARF, AS JDR, AW, AL and HH. BWF and HH have verified the underlying data. All authors have read and approved the final version and submission. The authors alone are responsible for the views expressed in this Article and they do not necessarily represent the views, decisions or policies of the bodies with which they are affiliated.

## Data sharing statement

Core and Extension Study underlying data have been disclosed as required by corresponding regulations and are publicly available on ClinicalTrials.gov, the EU Clinical Trial Registry, and the Novartis Clinical Trial Results websites. Novartis is committed to sharing with qualified external researchers, access to patient-level data and supporting clinical documents from eligible studies. These requests are reviewed and approved by an independent review panel on the basis of scientific merit. All data provided is anonymized to respect the privacy of patients who have participated in the trial in line with applicable laws and data availability is according to the criteria and process described on www.clinicalstudydatarequest.com.

## Declaration of interests

NM declares personal fees from InfanDx, AS declares personal fees from Novartis, Bayer, Allergan, Apellis and Recordati Rare Diseases, DL declares personal fees from Novartis and Bayer; ARF declares personal fees from Bayer and Recordati Rare Diseases, all outside the submitted work. AW is an employee of and owns stock from Novartis (Basel, Switzerland); HH is an employee of China Novartis Institutes for Biomedical Research (Shanghai, China); AL is an employee of Novartis Pharmaceuticals Corporation, East Hanover, New Jersey, USA.
