# Supplementary material for: Ranibizumab versus laser therapy for the treatment of very low birthweight infants with retinopathy of prematurity (RAINBOW): five-year outcomes of a randomised trial
Source: eClinicalMedicine. 2024 Apr 11;71:102567. doi: 10.1016/j.eclinm.2024.102567 (PMC11024572; doi:10.1016/j.eclinm.2024.102567)

Supplemental information for:

Ranibizumab versus laser therapy for the treatment of very low birthweight infants with retinopathy of prematurity (RAINBOW): Five-year outcomes of a randomised trial.

Marlow et al 2023

Contents:

Supplemental Table 1: Investigator reports of ocular adverse events (including serious (SAE) and other AE) reported from enrolment into RAINBOW Core Study up to 5 years of age (where not reported in main report).

Supplemental Table 2: Investigator reported systemic (non-ocular) serious adverse events (SAE) from enrolment into RAINBOW Core Study up to 5 years of age.

Supplemental Table 3: Investigator-identified non-ocular adverse events (including SAE and AE) relating to neurodevelopment in children enrolled in RAINBOW Extension Study.

Supplemental Figure 1: Cycloplegic refractive state at 5 years of age for children evaluated in the RAINBOW extension study.

Supplemental Figure 2: Children's Visual Function Questionnaire ratings for total and subscales among children evaluated at 5 years.

Supplemental Figure 3: Height and weight z-scores at 5 years in three RAINBOW trial groups

Supplemental table 1: Investigator reports of ocular adverse events (serious (SAE) and other AE) reported from enrolment into RAINBOW Core Study up to 5 years of age (where not reported in main report)

Primary system organ class<br>Preferred term	Ranibizumab 0·2mg<br>n=61	Ranibizumab 0·1mg<br>n=65	Laser<br>n=64
**Serious Adverse Events Total (n (%) children)**	**0**	**6 (9%)**	**2 (4%)**
**Eye disorders**	**0**	**4 (6%)**	**2 (4%)**
Conjunctival haemorrhage	-	-	1
Eye disorder	-	1	-
Retinal detachment	-	2	0
Retinal haemorrhage	-	-	1
Retinopathy of prematurity	-	1	1
**Infections and infestations**	**-**	**1 (2%)**	**-**
Periorbital cellulitis	-	1	-
**Injury, poisoning and procedural complications**	**-**	**-**	**1 (2%)**
Periorbital haematoma	-	-	1
**Adverse Events Total (n (%) children)**	**19 (31%)**	**26 (40%)**	**22 (41%)**
**Eye disorders**	**16 (26%)**	**22 (34%)**	**20 (37%)**
**Disorders in table 2 plus:**			
Astigmatism	3	2	2
Hypermetropia	2	1	-
Amblyopia	1	2	1
Heterophoria	1	-	-
Swelling of eyelid	1	-	-
Amblyopia strabismic	-	-	1
Anisometropia	-	1	2
Atrophy of globe	-	1	-
Blepharitis	-	1	-
Cataract	-	1	-
Conjunctival haemorrhage	-	-	1
Conjunctival hyperaemia	-	1	-
Conjunctivitis allergic	-	1	-
Corneal scar	-	1	-
Eccentric fixation	-	1	-
Exposure keratitis	-	1	-
Eye disorder	-	1	-
Eyelid oedema	-	1	-
Lenticular opacities	-	1	-
Maculopathy	-	1	-
Orbital oedema	-	1	-
Refractive amblyopia	-	-	1
Retinal detachment	-	2	-
Retinal haemorrhage	-	-	1
Vitreous haemorrhage	-	1	-
**Infections and infestations**	**2 (3%)**	**6 (9%)**	**4 (7%)**
Conjunctivitis	2	4	4
Bacterial infection	-	-	1
Cellulitis	-	1	-
Eye infection	-	-	1
Periorbital cellulitis	-	1	-
Rash pustular	-	1	-
Rhinitis	-	1	-
**Congenital, familial and genetic disorders**	**-**	**1 (2%)**	**-**
Dacryostenosis congenital	-	1	-
**Immune system disorders**	**1 (2%)**	**-**	**-**
Seasonal allergy	1	-	-
**Injury, poisoning and procedural complications**	**1 (2%)**	**-**	
Eyelid injury	1	-	-

Notes:

- MedDRA version 25.0 was used for reporting.
- A subject with multiple occurrences of an AE for a preferred term or system organ class is counted only once in each specific category.
- Primary system organ classes are presented alphabetically; preferred terms are sorted within primary system organ class by descending frequency in the ranibizumab 0·2 mg arm.

Supplemental table 2: Investigator reported systemic (non-ocular) serious adverse events (SAE) from enrolment into RAINBOW Core Study up to 5 years of age.

Primary system organ class<br>Preferred term	Ranibizumab 0·2mg<br>n=61	Ranibizumab 0·1mg<br>n=65	Laser<br>n=64
**Adverse Events Total (n (%) children)**	**21 (34%)**	**23 (35%)**	**25 (46%)**
**Infections and infestations**	**14 (23%)**	**16 (25%)**	**18 (33%)**
Bronchiolitis	4	3	0
Bronchitis	3	3	5
Pneumonia	3	4	4
Upper respiratory tract infection	2	0	1
Croup infectious	1	0	0
Ear infection	1	0	1
Gastroenteritis	1	0	2
Hand-foot-and-mouth disease	1	0	0
Herpangina	1	0	0
Lower respiratory tract infection	1	0	0
Meningitis viral	1	0	0
Otitis media acute	1	0	1
Parainfluenzae viral bronchitis	1	0	0
Pharyngitis	1	2	1
Pyelonephritis	1	0	0
Respiratory syncytial virus bronchiolitis	1	1	2
Respiratory syncytial virus bronchitis	1	1	0
Respiratory syncytial virus infection	1	0	1
Adenovirus infection	0	1	0
Bacterial food poisoning	0	0	1
Bronchitis viral	0	1	0
Cellulitis	0	1	0
Enterocolitis infectious	0	1	0
Epstein-Barr virus infection	0	0	1
Gastroenteritis adenovirus	0	0	1
Gastroenteritis norovirus	0	0	1
Gastroenteritis rotavirus	0	1	1
Gastroenteritis viral	0	0	1
Influenza	0	0	1
Laryngitis	0	1	0
Leptospirosis	0	0	1
Pharyngitis streptococcal	0	1	0
Pneumonia aspiration	0	0	1
Pneumonia respiratory syncytial viral	0	2	0
Pneumonia viral	0	0	1
Respiratory tract infection viral	0	0	1
Septic shock	0	0	1
Streptococcal infection	0	0	1
Tonsillitis	0	0	1
Viral infection	0	0	1
Viral pharyngitis	0	1	0
Viral tonsillitis	0	1	0
Viral upper respiratory tract infection	0	1	0
**Gastrointestinal**	**3 (5%)**	**8 (12%)**	**5 (9%)**
Constipation	2	1	1
Enterocolitis	1	0	0
Flatulence	1	0	0
Ileus	1	0	0
Vomiting	1	3	2
Abdominal discomfort	0	1	0
Acetonaemic vomiting	0	1	0
Diarrhoea	0	1	0
Gastrointestinal haemorrhage	0	1	0
Gastrooesophageal reflux disease	0	0	1
Haematemesis	-	-	1
Haematochezia	-	-	1
Inguinal hernia	-	-	1
Mechanical ileus	-	1	-
Necrotising colitis	-	-	1

Primary system organ class<br>Preferred term	Ranibizumab 0·2mg<br>n=61	Ranibizumab 0·1mg<br>n=65	Laser<br>n=64
**Adverse Events Total (n (%) children)**	**21 (34%)**	**23 (35%)**	**25 (46%)**
**General Disorders**	**5 (8%)**	**1 (2%)**	**3 (6%)**
Pyrexia	3	1	2
Developmental delay	2	-	-
Inflammation	-	-	1
**Metabolism and nutrition**	**6 (1-%)**	**2 (3%)**	**1 (2%)**
Dehydration	3	-	1
Decreased appetite	1	-	-
Hyperphosphatasaemia	1	-	-
Hypophagia	1	-	-
Electrolyte imbalance	-	1	-
Failure to thrive	-	1	-
Hypernatraemia	-	1	-
**CNS disorders**	**4 (7%)**	**5 (8%)**	**2 (4%)**
Febrile convulsion	2	3	-
Cerebellar haemorrhage	1	-	-
Hydrocephalus	1	-	1
Myoclonus	1	-	-
Cognitive disorder	-	1	-
Epilepsy	-	-	1
Intracranial pressure increased	-	1	-
Motor dysfunction	-	1	-
Periventricular leukomalacia	-	1	-
Quadriparesis	-	1	-
**Respiratory or thoracic**	**6 (1-%)**	**6 (9%)**	**5 (9%)**
Laryngospasm	2	-	-
Asthma	1	3	2
Bronchial obstruction	1	-	-
Pneumonitis	1	-	-
Pulmonary hypertension	1	-	-
Acute respiratory failure	-	2	-
Bronchopulmonary dysplasia	-	-	1
Bronchospasm	-	-	1
Cough	-	-	1
Dyspnoea	-	1	-
Hypercapnia	-	1	-
Hypoxia	-	2	-
Increased bronchial secretion	-	1	-
Nasal discharge discolouration	-	1	-
Pulmonary vein stenosis	-	1	-
Respiratory distress	-	1	1
Respiratory failure	-	2	-
Tonsillar hypertrophy	-	-	1
**Cardiac disorders**	**1 (2%)**	**1**	**1**
Bradycardia	1	1	-
Cardiac arrest	-	-	1
**Congenital, familial and genetic disorders**	**-**	**2 (3%)**	**1**
Cerebral palsy	-	1	-
Coarctation of the aorta	-	-	1
Cryptorchism	-	1	-
**Ear and labyrinth disorders**	**1 (2%)**	**-**	**-**
Deafness	1	-	-
**Endocrine disorders**	**-**	**-**	**1**
Diabetes insipidus	-	-	1
**Hepatobiliary disorders**	**-**	**3 (5%)**	**-**
Cholelithiasis	-	1	-
Hepatic failure	-	1	-
Portal hypertension	-	1	-
**Injury, poisoning and procedural complications**	**1 (2%)**	**1 (2%)**	**1 (2%)**
Head injury	1	-	-
Endotracheal intubation complication	-	1	-
Foreign body in respiratory tract	-	-	1
**Investigations**	**1 (2%)**	**1 (2%)**	**-**
Oxygen saturation decreased	1	1	-
**Musculoskeletal and connective tissue disorders**	**-**	**-**	**1 (2%)**

Primary system organ class<br>Preferred term	Ranibizumab 0·2mg<br>n=61	Ranibizumab 0·1mg<br>n=65	Laser<br>n=64
**Adverse Events Total (n (%) children)**	**21 (34%)**	**23 (35%)**	**25 (46%)**
Osteopenia	-	-	1
**Neoplasms benign, malignant and unspecified**	**1 (2%)**	**1 (2%)**	**-**
Haemangioma	1	1	-
**Pregnancy, puerperium and perinatal conditions**	**-**	**-**	**1 (2%)**
Perinatal brain damage	-	-	1
**Psychiatric disorders**	**-**	**1 (2%)**	**-**
Psychomotor retardation	-	1	-
**Renal and urinary disorders**	**1 (2%)**	**1 (2%)**	**-**
Nephrolithiasis	1	-	-
Acute kidney injury	-	1	-
**Skin and subcutaneous tissue disorders**	**1 (2%)**	**2 (3%)**	**-**
Erythema multiforme	1	-	-
Urticarial vasculitis	1	-	-
Rash	-	1	-
Rash papular	-	1	-

Notes:

- MedDRA version 25.0 was used for reporting.
- A subject with multiple occurrences of an AE for a preferred term or system organ class is counted only once in each specific category.
- Primary system organ classes are presented alphabetically; preferred terms are sorted within primary system organ class by descending frequency in the ranibizumab 0·2 mg arm.
- Investigator led reporting – may be reported elsewhere in the report as secondary outcomes.

Supplemental Table 3: Investigator-identified non-ocular adverse events (AE) relating to neurodevelopment in children enrolled in RAINBOW Extension Study. Serious and other AEs that start during core study and are ongoing at extension baseline or start on/after extension baseline are included. Multiple occurrences of the same event in a patient were counted only once. MedDRA v25.0 was used for description of safety concerns, v25.0 for coding of study AEs. Percentages are based on the number of patients in the Safety set in the specific treatment group.

	**Ranibizumab 0·2mg N=61 n (%)**	**Ranibizumab 0·1mg n=65 n (%)**	**Laser n=64 n (%)**
**Neurodevelopmental**	**10 (16·4)**	**6 (9·2)**	**9 (16·7)**
Cerebral palsy	0	3 (4·6)	2 (3·7)
Cognitive disorder	0	1 (1·5)	0
Deafness	3 (4·9)	0	1 (1·9)
Deafness bilateral	0	0	1 (1·9)
Deafness transitory	1 (1·6)	0	0
Developmental coordination disorder	0	1 (1·5)	1 (1·9)
Developmental delay	7 (11·5)	1 (1·5)	3 (5·6)
Hypoacusis	0	0	1 (1·9)
Intellectual disability	0	0	1 (1·9)
Motor developmental delay	2 (3·3)	0	0
Motor dysfunction	0	1 (1·5)	1 (1·9)
Psychomotor retardation	0	1 (1·5)	0
Psychomotor skills impaired	0	1 (1·5)	0
Speech disorder developmental	1 (1·6)	2 (3·1)	0

Supplemental Figure 1: Cycloplegic refractive state at 5 years of age for children evaluated in the RAINBOW extension study. The threshold for high myopia used was –5D (dashed line); solid bars indicate median and interquartile ranges.

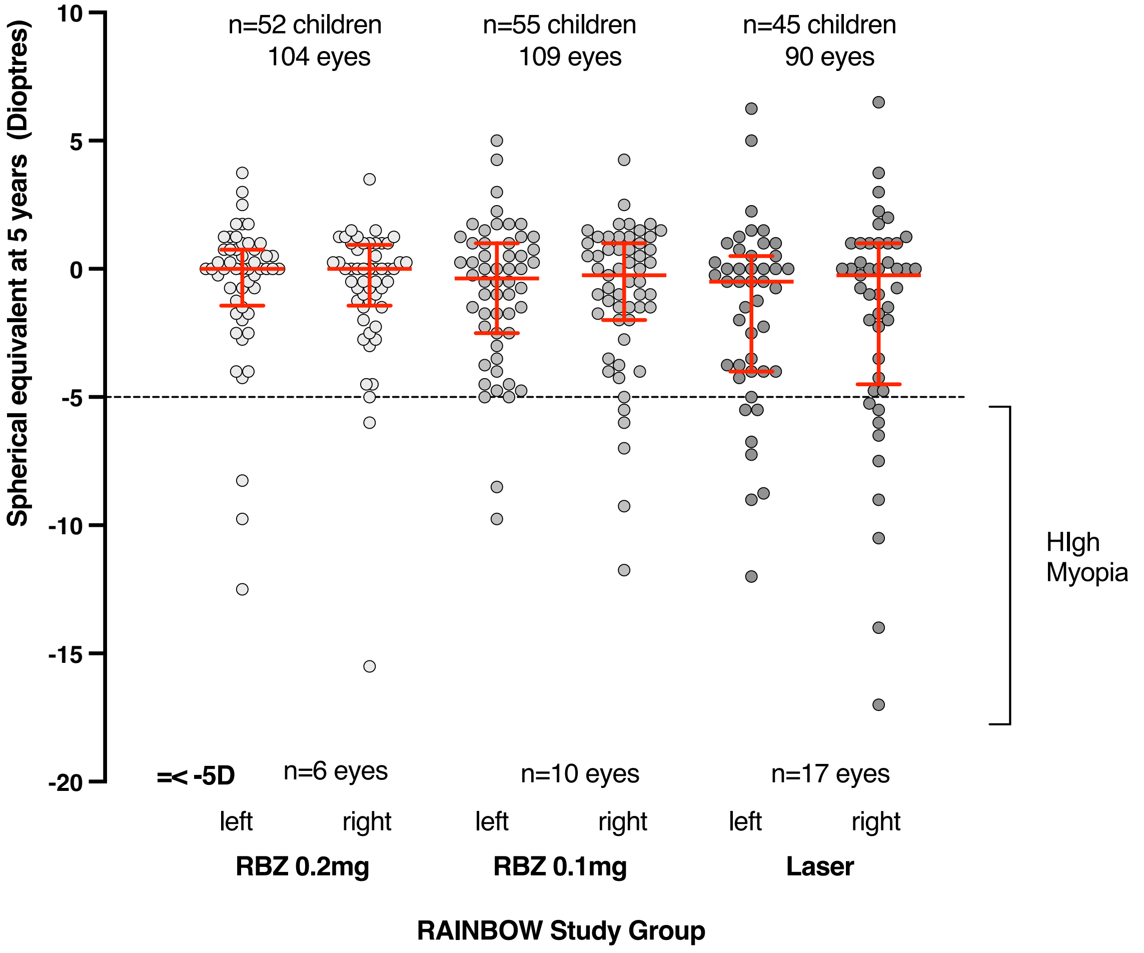

Supplemental Figure 2: Children's Visual Function Questionnaire ratings for total and subscales among children evaluated at 5 years. Data are from 52 children following RBZ 0·2mg, 55 children following RBZ 0·1mg and 45 children following laser; please note that the treatment scale was only completed for those who have received any form of eye treatment (for example glasses, amblyopia treatment, strabismus surgery) in the extension study. Boxes represent median and 25th and 75th percentiles, whiskers 5th and 95th percentiles, and circles represent outlying data points.

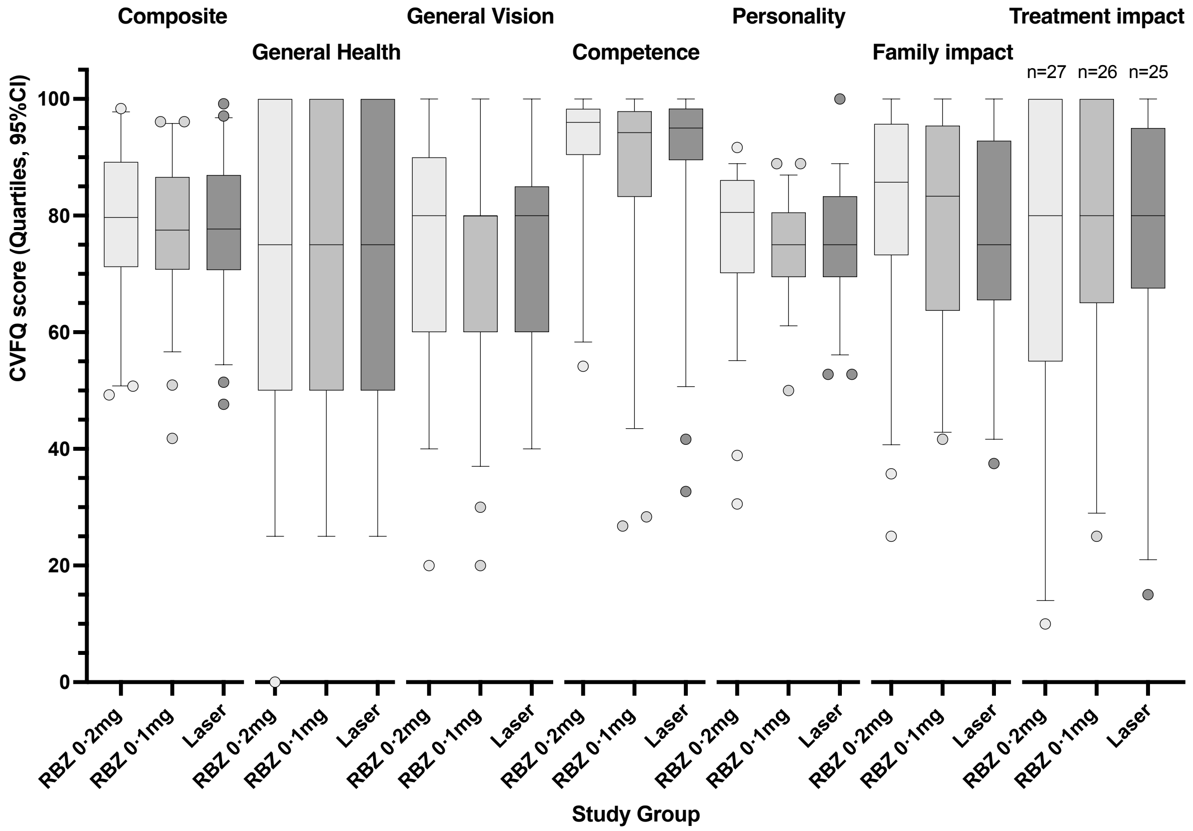

Supplemental Figure 3: Height and weight z-scores at 5 years in three RAINBOW trial groups. Horizontal bar indicates mean values in each study group.

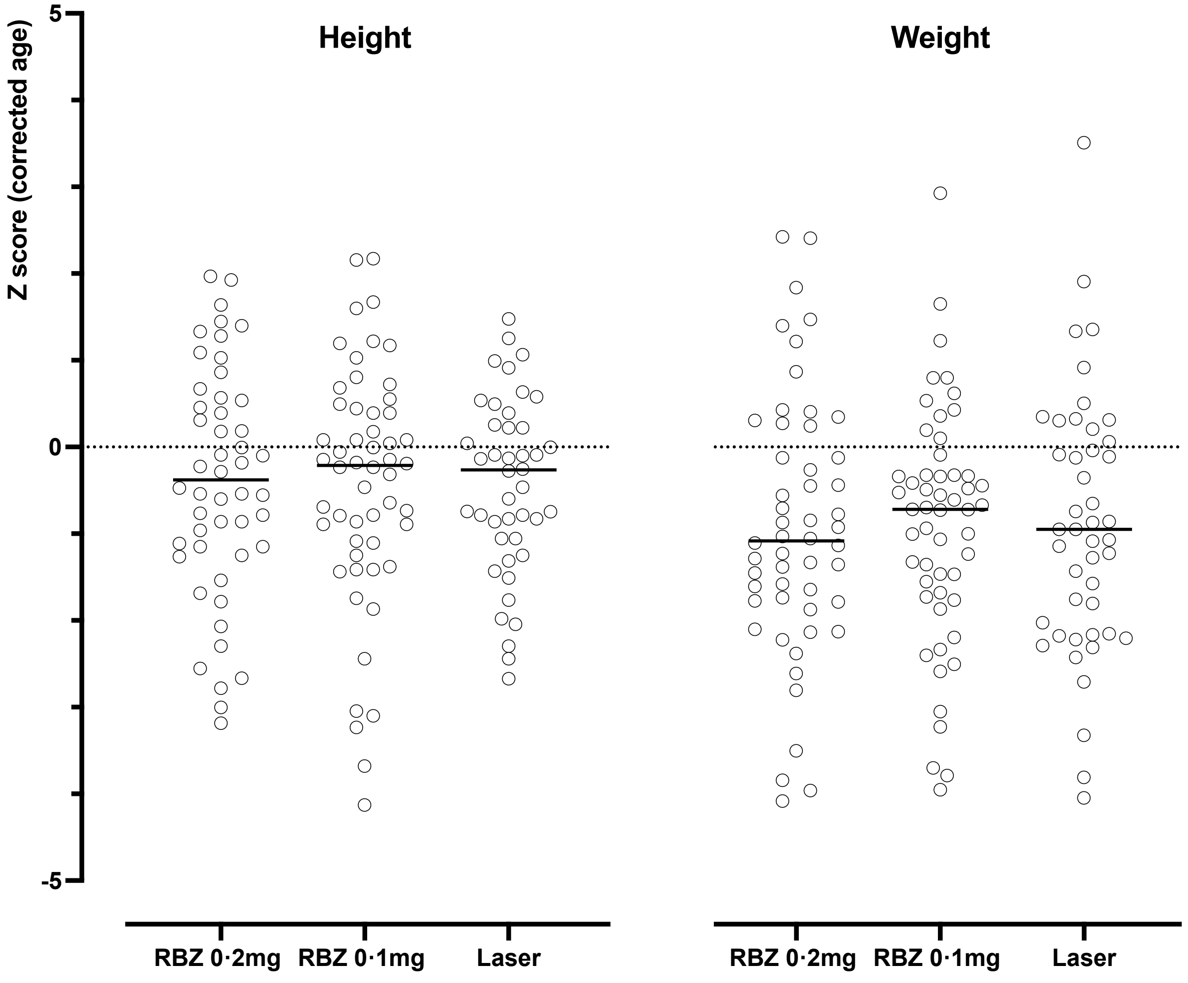

Supplement: Supplemental data [file mmc1.pdf]
