# Supplementary material for: Ranibizumab versus laser therapy for the treatment of very low birthweight infants with retinopathy of prematurity (RAINBOW): five-year outcomes of a randomised trial
Source: eClinicalMedicine. 2024 Apr 11;71:102567. doi: 10.1016/j.eclinm.2024.102567 (PMC11024572; doi:10.1016/j.eclinm.2024.102567)
Supplement: Prot_000.pdf [file mmc2.pdf]

Global Clinical Development - General Medicine

RFB002/Ranibizumab

Clinical Trial Protocol CRFB002H2301E1 / NCT02640664

RAINBOW extension study: an extension study to evaluate the long term efficacy and safety of RAnibizumab compared with laser therapy for the treatment of INfants BOrn prematurely With retinopathy of prematurity

Document type:	Amended Protocol Version
EUDRACT number:	2014-004048-36 (PIP number: 000527-PIP04-13)
Version number:	v02 (Clean)
Clinical trial phase:	III
Release date:	10-Jul-2019

Property of Novartis
Confidential
May not be used, divulged, published, or otherwise disclosed
without the consent of Novartis

Clinical Trial Protocol Template Version 03 (August 2015)

[redacted]

Table of contents

Table of contents 2
List of tables 4
List of figures 5
List of abbreviations 6
Glossary of terms....................8
Amendment 2 9
Protocol summary....................11
1 Introduction 15
1.1 Background....................15
1.1.1 Ranibizumab 16
1.2 Purpose 19
2 Study objectives and endpoints 19
2.1 Primary objective(s)....................19
2.2 Secondary objective(s)....................19
[redacted] 19
2.4 Objectives and related endpoints....................19
3 Investigational plan 25
3.1 Study design....................25
3.2 Rationale for study design 25
3.3 Rationale for dose/regimen, route of administration and duration of treatment....26
3.4 Rationale for choice of comparator 26
3.5 Purpose and timing of interim analyses/design adaptations....................26
3.5.1 Interim analysis 1 26
3.5.2 Interim analysis 2 27
3.6 Risks and benefits....................27
4 Population....................29
4.1 Inclusion criteria 29
4.2 Exclusion criteria....................30
5 Treatment....................30
5.1 Study treatment....................30
5.1.1 Investigational and control drugs....................30
5.1.2 Additional treatment....................30
5.2 Treatment arms 30
5.3 Treatment assignment and randomization....................31
5.4 Treatment blinding....................31

[redacted]

5.5 Treating the patient ... 31
5.5.1 Patient numbering ... 31
5.5.2 Dispensing the study drug ... 31
5.5.3 Handling of study and additional treatment ... 31
5.5.4 Instructions for prescribing and taking study treatment ... 32
5.5.5 Permitted dose adjustments and interruptions of study treatment ... 33
5.5.6 Rescue medication ... 33
5.5.7 Concomitant medication ... 34
5.5.8 Prohibited medication ... 34
5.5.9 Emergency breaking of assigned treatment code ... 34
5.6 Study Completion and Discontinuation ... 35
5.6.1 Study completion and post-study treatment ... 35
5.6.2 Discontinuation of Study Treatment ... 35
5.6.3 Premature patient withdrawal from the study ... 35
5.6.4 Loss to follow-up ... 36
5.6.5 Early study termination by the sponsor ... 36
6 Visit schedule and assessments ... 36
6.1 Information to be collected on screening failures ... 42
6.2 Patient demographics/other baseline characteristics ... 42
6.3 Treatment exposure and compliance ... 42
6.4 Efficacy ... 42
6.4.1 Ophthalmic fundus features ... 42
6.4.2 Ocular and visual function ... 43
6.4.3 Visual Acuity (VA) ... 43
6.4.4 Appropriateness of efficacy assessments ... 44
6.5 Safety ... 44
6.5.1 Ocular examination ... 44
6.5.2 Vital signs ... 44
6.5.3 Health status ... 45
6.5.4 Laboratory evaluations ... 45
6.5.5 Appropriateness of safety measurements ... 45
[redacted] 46
[redacted] 46
7 Safety monitoring ... 46
7.1 Adverse events ... 46
7.2 Serious adverse events ... 48

[redacted]

7.2.1 Definition of SAE 48
7.2.2 SAE reporting....49
7.3 Reporting of study treatment errors....50
8 Data review and database management....51
8.1 Site monitoring 51
8.2 Data collection....51
8.3 Database management and quality control....51
8.4 Data Monitoring Committee....52
8.5 Adjudication Committee....52
9 Data analysis....52
9.1 Analysis sets 53
9.2 Patient demographics and other baseline characteristics....53
9.3 Treatments (study drug, rescue medication, other concomitant therapies, compliance)....53
9.4 Analysis of the primary variable(s) 54
9.4.1 Variable(s)....54
9.4.2 Statistical model, hypothesis, and method of analysis....54
9.4.3 Handling of missing values/censoring/discontinuations....55
9.4.4 Sensitivity analyses 55
9.5 Analysis of secondary variables 55
9.5.1 Efficacy variables....55
9.5.2 Safety variables 56
[redacted] 57
9.7 Interim analyses 57
9.8 Sample size calculation....59
10 Ethical considerations....59
10.1 Regulatory and ethical compliance....59
10.2 Informed consent procedures....59
10.3 Responsibilities of the investigator and IRB/IEC....59
10.4 Publication of study protocol and results....59
11 Protocol adherence 60
11.1 Protocol Amendments 60
12 References 60

List of tables

Table 2-1 Objectives and related endpoints 20

[redacted]

Table 5-1 Study ranibizumab dose in Extension study based on treatment received at the baseline visit in core study (CRFB002H2301) 33
Table 5-2 Treatment which prohibits further use of study ranibizumab 34
Table 6-1 Main Assessment Schedule 38
Table 6-2 Additional Assessment Schedule – Treatment with Ranibizumab 41

List of figures

Figure 1-1 Predicted Systemic Exposure After Anti-VEGF Treatment, Classified Based on Toxicological Outcomes....................................... 18
Figure 3-1 Study design 25

[redacted]

List of abbreviations

AE	Adverse Event
ALP	Alkaline Phosphatase
ALT	Alanine Aminotransferase
ANOVA	Analysis of variance
AST	Aspartate Aminotransferase
ATC	Anatomical Therapeutic Chemical (classification system)
BEAT-ROP	Bevacizumab Eliminates the Angiogenic Threat of Retinopathy of Prematurity (study)
BUN	Blood urea nitrogen
CFR	US Code of Federal Regulations
CPO	Country Pharma Organization
CRF	Case Report/Record Form (paper or electronic)
CTT	Clinical Trial Team
[redacted]	[redacted]
DMC	Data Monitoring Committee
DS&E	Drug Safety & Epidemiology
eCRF	electronic Case Report/Record Form
EMA	European Medicines Agency
EEA	European Economic Area
ETDRS	Early Treatment Diabetic Retinopathy Study
ETROP	Early Treatment for Retinopathy of Prematurity (study)
EU	European Union
GCP	Good Clinical Practice
GDPR	General Data Protection Regulation
[redacted]	[redacted]
Hb	Hemoglobin
IB	Investigator’s Brochure
ICH	International Conference on Harmonization of Technical Requirements for Registration of Pharmaceuticals for Human Use
IEC	Independent Ethics Committee
Ig	Immunoglobulin
IRB	Institutional Review Board
IRT	Interactive Response Technology
MedDRA	Medical dictionary for regulatory activities
OC/RDC	Oracle Clinical/Remote Data Capture
PIP	EU Pediatric Investigation Plan
PLT	Platelet count
ROP	Retinopathy of Prematurity
SAE	Serious Adverse Event
SAP	Statistical Analysis Plan
SmPC	Summary of Product Characteristics
SUSAR	Suspected Unexpected Serious Adverse Reactions
VA	Visual Acuity

[redacted]

VEGF	Vascular endothelial growth factor
WCC	White Cell Count
WHO	World Health Organization
WoC	Withdrawal of Consent

[redacted]

Glossary of terms

Assessment	A procedure used to generate data required by the study
Chronological age	A child’s chronological age is its age referring to the date of birth
Control drug	Drugs(s) used as a comparator to reduce assessment bias, preserve blinding of investigational drug, assess internal study validity, and/or evaluate comparative effects of the investigational drug
Corrected age	A child’s corrected age is its chronological age corrected for its prematurity, i.e. calculated by subtracting the number of weeks of prematurity from the chronological age
Dosage	Dose of the study treatment given to the patient in a time unit (e.g. 100 mg once a day, 75 mg twice a day)
Enrollment	Point/time of patient entry into the study at which informed consent must be obtained (e.g. prior to starting any of the procedures described in the protocol)
Epoch	A portion of the study which serves a specific purpose. Typical epochs are: screening/recruitment, wash-out, treatment, and follow-up
Investigational drug	The drug whose properties are being tested in the study; this definition is consistent with US CFR 21 Section 312.3 and is synonymous with “investigational new drug” or “investigational medicinal product.”
Medication pack number	A unique identifier on the label of each investigational drug package
Patient/subject ID	A unique number assigned to each patient upon signing the informed consent
Personal Data	Subject information collected by the Investigator that is transferred to Novartis for the purpose of the clinical trial. This data includes subject identifier information, study information and biological samples.
Randomization number	A unique identifier assigned to each randomized patient, corresponding to a specific treatment arm assignment
Study drug/ treatment	Any single drug or combination of drugs administered to the patient as part of the required study procedures; includes investigational drug (s), placebo/comparator active drug run-ins or background therapy
Study Treatment Discontinuation	When the investigator permanently stops to administer study treatment to the patient prior to the defined study treatment completion date
Variable	A measured value or assessed response that is determined in specific assessments and used in data analysis to evaluate the drug being tested in the study
Withdrawal of study consent (WoC)	Withdrawal of consent from the study occurs only when a patient’s parent(s) or legal guardian(s) does not want the patient to participate in the study any longer and does not allow any further collection of personal data.

[redacted]

Amendment 2

Amendment rationale

The main purpose of this amendment is to:

- Include the requirement for masked VA assessments to be performed at the child's 5th birthday visit following a Health Authority request.

Changes to the protocol

Major changes are made to the protocol in the following sections:

- Protocol summary and Section 3.1 "Study Design": The sections were amended to update language that the VA assessor conducting the child's 5th year birthday visit will be masked to treatment.
- Section 5.4 "Treatment Blinding": The section was amended to update the study design open label language and clarify that the VA assessor conducting the child's 5th year birthday visit will be masked to treatment.
- Section 6.4.3 "Visual Acuity (VA)": The section was amended to require a masked assessor to perform VA assessments at the child's 5th birthday visit.

Changes to specific sections of the protocol are shown in the track changes version of the protocol using strike through red font for deletions and red underlined for insertions.

IRB/ IECs

A copy of this amended protocol will be sent to the Institutional Review Board (IRBs)/ Independent Ethics Committee (IECs) and Health Authorities.

The changes described in this amended protocol require IRB/ IEC and Health Authority approval according to local regulations prior to implementation.

The changes herein do NOT affect the trial specific global model consent form.

Summary of previous amendments

Amendment 1 (31-Jul-2018)

Amendment rationale

The main purpose of this amendment is to:

- Collect additional visual function data following EU national scientific advice meeting.

In addition, further purpose of this amendment is to:

- Update the primary hypothesis testing to also include a scenario where the primary efficacy variable of the core study (CRFB002H2301) does not meet the pre-defined statistical significance level: In this case, the primary objective will only be analyzed using descriptive statistics.

Changes to the protocol

Major changes are made to the protocol in the following sections:

[redacted]

- Section 6 “Visit schedule and assessments”: A supplemental visit and additional vision tests at the child’s 2 and 3 years’ corrected age visits were added to assess visual function.
- “Protocol summary”, Section 9.4 “Analysis of the primary variable(s)” and Section 9.5 “Analysis of secondary variables”: Wording updated to reflect the change in the analysis of the primary objective

Other changes are made to the protocol in the following sections:

Section 5.6.3.1 “Withdrawal of informed consent” was updated to reflect the new requirements of the General Data Protection Regulation (GDPR) within the European Economic Area (EEA).

Section 6.4.3 “Visual Acuity” was updated, details of VA assessment were removed for clarity.

In addition, this amendment incorporates editorial changes to the protocol language to enhance consistency and clarity throughout the protocol.

Changes to specific sections of the protocol are shown in the track changes version of the protocol using strike through red font for deletions and red underlined for insertions.

IRB/ IECs

A copy of this amended protocol will be sent to the Institutional Review Board (IRBs)/ Independent Ethics Committee (IECs) and Health Authorities.

The changes described in this amended protocol require IRB/ IEC and Health Authority approval prior to implementation.

The changes herein affect the Informed Consent. Sites are required to update and submit for approval a revised Informed Consent that takes into account the changes described in this protocol amendment.

[redacted]

Protocol summary

**Protocol number**	CRFB002H2301E1
**Title**	RAINBOW extension study: an extension study to evaluate the long term efficacy and safety of RAnibizumab compared with laser therapy for the treatment of INfants BOrn prematurely With retinopathy of prematurity
**Brief title**	RAINBOW extension study
**Sponsor and Clinical Phase**	Novartis; Phase 3
**Investigation type**	Drug
**Study type**	Interventional
**Purpose and rationale**	The purpose of this study is to evaluate the long term efficacy and safety of intravitreal ranibizumab compared with laser ablation therapy in patients who were treated for retinopathy of prematurity (ROP) in the core study CRFB002H2301
**Primary Objective(s)**	To evaluate the visual function of patients, by assessing the visual acuity in the better-seeing eye at the patient's fifth birthday
**Secondary Objectives**	• To evaluate the safety outcomes by analyzing the type, frequency and severity of ocular and non-ocular Adverse Events (AEs)<br>• To evaluate the visual function by assessing the visual acuity in the worse-seeing eye at the patient's fifth birthday<br>• To evaluate the absence of active ROP at 40 weeks and 52 weeks post baseline visit in the core study<br>• To evaluate the absence of ocular structural abnormalities at 40 weeks post baseline visit in the core study, at the patient's 2 years corrected age and fifth birthday<br>• To evaluate the recurrence of ROP up to 40 weeks and 52 weeks post baseline visit in the core study<br>• To assess the number of ranibizumab injections received in the treatment of patients with ROP up to 40 weeks post baseline visit in the core study<br>• To evaluate the refraction in each eye at the patient's 2 years' corrected age and fifth birthday<br>• To evaluate the physical development at the patient's 2 years' corrected age and fifth birthday<br>• To evaluate the health status at the patient's 2 years' corrected age and fifth birthday (respiratory function, hearing function, duration of hospitalization, and weight at discharge from hospital)
**Study design**	This is a multicenter open-label extension study where the VA assessment at the child's 5th birthday visit will be performed by an assessor who is masked to study treatment. Patients who have successfully completed the 24-week core study CRFB002H2301 are eligible for entry into the study.<br>Treatment with study ranibizumab will be permitted for eligible eyes up to and including week 40 from the baseline visit in the core study (CRFB002H2301) [Epoch 1]. The remainder of the extension study [Epoch 2] is observational.
**Population**	The study population will consist of male and female preterm infants/children who have successfully completed the CRFB002H2301

[redacted]

	core study. These patients received intravitreal ranibizumab and/or laser therapy for retinopathy of prematurity in the core study.
**Key Inclusion criteria**	Patients have to fulfill all of the following criteria at baseline of the extension study prior to receiving the first study related procedure:<br>1. Signed informed consent from parent(s) or legal guardian(s), in compliance with local requirements<br>2. The patient successfully completed the core study CRFB002H2301, as defined by providing assessments at the Visit 112 or, if appropriate, at the last of the additional assessment visits as per protocol in CRFB002H2301, whichever is latest<br>3. The patient received study treatment in both eyes at baseline of study CRFB002H2301
**Key Exclusion criteria**	Patients fulfilling any of the following criteria at study entry are not eligible for inclusion in this study:<br>1. Patient has a medical condition or personal circumstance which precludes study participation or compliance with study procedures, as assessed by the Investigator<br>2. Patient has been discontinued from the core study CRFB002H2301 at any time
**Study treatment**	Ranibizumab 10 mg/mL solution for injection (labeled as "RFB002 0.5 mg/0.05 mL") supplied in glass vials for single use by Novartis Drug Supply Management. The storage conditions will be described on the medication label.
**Efficacy assessments**	Ophthalmic fundus features<br>These will be assessed in both eyes by indirect ophthalmoscopy:<br>• Features of active ROP disease:<br>• ROP disease – zone, stage, extent by clock hours, and characteristics of vascular changes of plus disease (extent by quadrants and severity)<br>• Extra-retinal vessels judged to be active<br>• Features of late sequelae of ROP/ ocular structural abnormalities:<br>• Retrolental membrane obscuring the view of the posterior pole<br>• Substantial temporal retinal vessel dragging causing abnormal structural features<br>• Posterior retinal fold involving the macula<br>• Retinal detachment involving the macula<br>• Retinal detachment not involving the macula<br>• Pre-retinal fibrosis<br>• Optic disc pallor<br>• Optic disc swelling<br>• Pigmentary disturbance in the macula<br>• Atrophic changes in macula<br>[redacted]<br>Ocular and visual function: refraction<br>Visual acuity (VA)<br>• Assessment of VA in each eye

**Key safety assessments**	• Ocular examination<br>• Standing/sitting height, weight, head circumference<br>• Adverse event monitoring (ocular and systemic); number of hospitalization/ prolongation due to Serious Adverse Event (SAE)<br>• Health status (respiratory function, hearing function, duration of hospitalization and weight at first discharge home)
[redacted]	
**Data analysis**	Three planned analyses will be conducted in the study.<br>• Interim analysis 1 will provide descriptive statistics for a subset of patients to evaluate ocular structural abnormalities. This is to comply with the plan for specific follow-up in the Pediatric Investigation Plan.<br>• Interim analysis 2 will be conducted on all patients at their 2 years' corrected age to report on the progress of the study to the scientific community.<br>• The final analysis will be conducted at the completion of the study.<br>Additional interim analyses may be conducted on safety or efficacy data as required.<br>All analyses will be carried out on the Extension Safety Set and displayed by the original study treatment received in the core study CRFB002H2301 unless otherwise specified. One-sided p-values and two sided 95% confidence intervals will be displayed without adjusting for multiplicity.<br>If the primary hypothesis testing of the core study shows statistical significance, the primary objective of the extension study will be assessed by testing for the superiority of ranibizumab 0.2 mg to laser of the visual acuity for the better-seeing eye at the patient's fifth birthday. An analysis of variance, with treatment group as a factor, stratified by the ROP zone defined at baseline in the core study will be performed using the Cochran-Mantel-Haenszel test. A two-way analysis of variance with treatment group and zone as factors will be used to estimate least squares means and 95% confidence intervals for visual acuity in the better-seeing eye within and between treatment groups. If statistical significance in the core study cannot be shown, only descriptive statistics will be provided for the comparison between ranibizumab 0.2 mg and laser.<br>The same approach will be used for inference conducted on continuous secondary efficacy variables. Inference conducted on binary secondary efficacy variables will use the Cochran-Mantel-Haenszel test for proportions stratified by the ROP zone defined at baseline in the core study.

[redacted]

	Descriptive statistics will be used to summarize other variables. All data will be listed.
**Key words**	Open-label, long-term, extension study; intravitreal ranibizumab; laser ablation therapy; retinopathy of prematurity; preterm infants; RAINBOW

[redacted]

1 Introduction

1.1 Background

Retinopathy of prematurity (ROP) is a vasoproliferative pathologic process that occurs in the incompletely vascularized, developing retina of low birth-weight preterm neonates. The vascular changes of ROP may be mild and regress completely with time without major long term sequelae, or may increase in severity and lead to macular dragging, total retinal detachment, severe visual impairment and lifelong blindness (Hardy et al. 2004). ROP is a significant cause of blindness in children in both developed and developing countries with approximately 50,000 children blind from ROP worldwide (Gilbert 2008, Mintz-Hittner et al. 2011).

In the 1990s, the use of laser photocoagulation to ablate the peripheral avascular retina gained widespread acceptance as standard of care treatment for ROP (Connolly et al 2002, Ng et al. 2002, Houston et al. 2013, Good 2004). However, despite improvement in the technology and timing of ROP treatment, ROP remains a leading cause of childhood blindness worldwide.

Laser therapy ablates the avascular peripheral retina and destroys the majority of the retinal cells that produce vascular endothelial growth factor (VEGF), which is postulated to play an important role in the initiation and progression of ROP (Smith 2008). Because of the role of VEGF in ROP, there is a growing body of evidence supporting the use of targeted pharmacologic inhibition of VEGF in the management of ROP (Sonmez et al. 2008, Sato et al. 2009).

Results from a prospective, multicenter, randomized clinical study (BEAT-ROP study) (Mintz-Hittner et al. 2011) suggest that intravitreal bevacizumab may be more efficacious than conventional laser ablation therapy among infants with stage 3+ ROP. The primary outcome of the study was the recurrence of retinal neovascularization before 54 weeks postmenstrual age that required re-treatment. It was found that among the subgroup of infants with zone I disease the recurrence rate was 6% with bevacizumab and 42% with conventional laser therapy. Moreover, bevacizumab therapy resulted in a mild anatomical retinal abnormality in only 1 eye of 31 infants, whereas conventional laser treatment resulted in a mild anatomical abnormality in 16 eyes, and a severe abnormality in 2 eyes, of 33 infants. Although the differences in outcomes were not statistically significant among infants with posterior zone II ROP, the results suggested a similar efficacy trend. An interesting finding of the BEAT-ROP study was that treatment with bevacizumab appeared to allow for continued vessel growth into the peripheral retina. In contrast, ablation therapy destroyed large areas of the peripheral retina and may affect associated vascular growth. If anti-VEGF agents allow more normal retinal vascularization to occur, it is anticipated that the rate of abnormal visual acuity, restricted visual fields, refractive errors, amblyopia, and strabismus may be reduced. This was demonstrated when patients from the BEAT-ROP study were assessed at a mean age of 2.5 years, with more myopia found in eyes that received laser treatment than in eyes that received intravitreal bevacizumab (Geloneck et al 2014).

A number of limitations have been noted in the BEAT-ROP study (Darlow et al. 2013). Therefore, despite reports of potential benefits of anti-VEGF in the treatment of ROP, a number of questions remain unanswered and require further study to better understand the clinical efficacy of anti-VEGF agents compared to standard of care laser therapy, the optimal dose to

[redacted]

be used, as well as more information on the long-term safety of intravitreal anti-VEGF use in a developing infant (Lee et al. 2011, Darlow et al. 2013). It is also important to collect more evidence on the longer term efficacy and safety outcomes in this patient group.

The study CRFB002H2301 is a randomized, open-label, three-arm parallel-group study to determine if intravitreal ranibizumab is superior to laser ablation therapy in the treatment of ROP. In addition, two doses of ranibizumab are investigated (0.1 mg and 0.2 mg). The study will assess the ability of these treatments to lead to regression of active ROP and prevention of the development of ocular complications that are associated with poor visual outcome 24 weeks after the first study treatment (intravitreal ranibizumab or laser ablation therapy).

This study, CRFB002H2301**E1**, is an extension study to evaluate the long term efficacy and safety of intravitreal ranibizumab compared with laser ablation therapy in ROP patients who successfully completed the core study (CRFB002H2301).

1.1.1 Ranibizumab

Ranibizumab is a recombinant humanized immunoglobulin (Ig) G1 kappa isotype monoclonal antibody fragment targeted against human VEGF-A.

Physical, Chemical, and Pharmaceutical Properties and Formulation

Detailed information on the physical, chemical and pharmaceutical properties of ranibizumab is described in the investigator’s brochure (IB).

Ranibizumab is available as a 10 mg/mL solution for intravitreal injection. Novartis is the marketing authorization holder for Lucentis® 10 mg/mL solution for injection in the European Union (EMEA/H/C/000715) and worldwide with the exception of the United States of America (US). The 10 mg/mL solution for injection is approved and commercialized globally.

The formulation is presented as a single-use sterile, clear to slightly opalescent, colorless to pale yellow and preservative free aqueous solution for intravitreal injection supplied in glass vials. All primary packaging materials are standard quality, suitable for packaging sterile liquid products, and comply with relevant pharmacopoeial requirements.

A syringe suitable to accurately deliver volumes of 10 µL and 20 µL of the solution into the eye will be used. This syringe will be a sterile, single-use disposable syringe intended for medical purposes. The syringe will be individually packaged. A standard injection needle (e.g., 30 G, ½ inch stainless steel) as for the adults will be used for the injection in premature neonates.

Neonatal Nonclinical and Clinical Information

The IB contains information on nonclinical studies of ranibizumab, as well as clinical studies in age-related macular degeneration, diabetic macular edema, retinal vein occlusion and pathologic myopia. Additional information relevant to the neonatal use of ranibizumab is described below.

Vascular endothelial growth factor plays an important angiogenic role in the embryo/fetus, during the postnatal period and in the adult life.

[redacted]

The 3 preclinical studies described below involved more extreme inhibition of systemic VEGF (e.g. transgenic mice and high dose of systemically administered VEGFR-1 decoy construct and VEGF-A antibody) than seen following intravitreal administration of a VEGF inhibitor.

Gerber et al 1999 reported on a conditional VEGF-knock out mouse model; incomplete/partial suppression of VEGF beginning at approximately Day 4 after birth was lethal to 38% of mice by Day 7. In surviving animals there was reported growth delays and liver maturation was delayed. Other organs evaluated (heart, kidney, lung and spleen) appeared small but normal. By Day 27 after birth, 10% of mice recovered body weight compared to wild type control mice.

Gerber et al. 1999 also dosed healthy newborn mice with either a mouse VEGFR-1 decoy construct (to deplete VEGF-A and VEGF-B), or an isotypic control protein. Weight loss was seen at doses as low as 1 mg/kg. The highest dose tested, 25 mg/kg, was lethal (deaths within 4-6 days of treatment). Target tissues identified were the fat stores (depleted), kidney (hemorrhage and altered growth), delayed growth of the lung and liver; and single-cell necrosis in the liver, heart, pancreas and spinal ganglia.

Malik et al. 2006 compared the effects of an anti-VEGF-A antibody and the mouse VEGFR-1 decoy construct in newborn mice at maximal pharmacodynamics doses. Both biologics decreased survival and weight-gain in the mice. Treatment delayed growth and vascularization of the liver, spleen, lung, heart, kidneys, and thymus.

Nonclinical toxicology data (ocular and systemic) on intravitreal administered ranibizumab were generated in cynomolgus monkeys that were 2 to 3.5 years old at study start, which is equivalent to adolescents/adults in terms of ocular and systemic organ development stage (Boothe et al 1985, Kiely et al 1987, Edward and Kaufman 2003, Qiao-Grider et al 2007). In monkeys, intravitreal administration of ranibizumab elicited essentially dose-dependent intraocular inflammatory responses that, at least in part, were considered to be an immune-mediated antibody response to a humanized protein, and thus of questionable relevance to humans. No systemic adverse effects were recorded in nonhuman primates administered up to 2 mg/eye for 26 weeks or when the intravitreal doses given to monkeys resulted in levels of systemically circulating ranibizumab that were higher than those observed in the clinical situation.

In pregnant cynomolgus monkeys, intravitreal administration of ranibizumab did not elicit developmental toxicity/teratogenicity and had no effect on weight/structure of the placenta. Bevacizumab or aflibercept administered intravenously to pregnant animals cause skeletal effects to the embryo/fetus (EMEA 2006, EMEA 2013). Therefore, the absence of embryo-fetal toxicity with ranibizumab is attributed to the very low systemic exposures after intravitreal administration and, potentially, from the inability of the antigen-binding fragment to cross the placental barrier due to lack of a crystallizable fragment region (unless anti-ranibizumab antibodies form and act as carrier proteins, thereby enabling placental transfer via binding to the neonatal crystallizable fragment receptor). However, due to its pharmacological mode of action, ranibizumab must be regarded as potentially teratogenic and embryo-fetal toxic.

[redacted]

[redacted]

[redacted]

[redacted]

[redacted]

[redacted]

[redacted]

The dosing regimen proposed in the ROP clinical program is also comparable to the one currently used by physicians treating ROP (age ≥ 26 weeks) with unlicensed anti-VEGF agents such as bevacizumab. Laser therapy performed after bevacizumab administration has been

[redacted]

reported to increase systemic drug exposures by damaging the natural barrier that represents a full-thickness retina (Wu et al 2011). However, no systemic complication (cardiovascular, respiratory, neurologic or renal) attributable to bevacizumab treatment (0.40-1.25 mg/eye) has been recorded so far in premature neonates (Micieli et al 2009, Law et al 2010, Sahin et al 2013, Wu et al 2013).

1.2 Purpose

The purpose of this study is to evaluate the long term efficacy and safety of intravitreal ranibizumab compared with laser ablation therapy in patients who were treated for ROP in the core study CRFB002H2301. All patients who successfully completed the CRFB002H2301 study will be invited to participate in this extension study CRFB002H2301**E1**.

The first study interim analysis will be conducted to comply with the plan for specific follow-up as agreed with the Pediatric Committee at the European Medicines Agency in the Pediatric Investigation Plan (see Section 3.2).

2 Study objectives and endpoints

All objectives will be assessed by the original study treatment received at baseline in the core study CRFB002H2301 unless otherwise specified.

2.1 Primary objective(s)

To evaluate the visual function of patients by assessing the visual acuity in the better-seeing eye at the patient's fifth birthday (see Table 2-1).

2.2 Secondary objective(s)

See Table 2-1.

[redacted]

2.4 Objectives and related endpoints

[redacted]

Table 2-1 Objectives and related endpoints

OBJECTIVE	Endpoint Title, Description and Reporting Time Frame for analysis and Unit of Measure	Stat Analysis Section
**Primary**		
To evaluate the visual function of patients, by assessing the visual acuity in the better-seeing eye at the patient's fifth birthday[a,e]	Title: visual acuity in the better-seeing eye at the patient's fifth birthday<br>Unit of Measure: visual acuity score (Lea symbols optotypes)<br>Description: see Section 6.4.3<br>Time Frame: at the patient's fifth birthday[e]	See Section 9.4
**Secondary**		
To evaluate the safety outcomes by analyzing the type, frequency and severity of ocular and non-ocular Adverse Events[b]	Title: ocular and non-ocular Adverse Events<br>Unit of Measure: number and percentage of patients having e.g. any AE; number of hospitalization/ prolongation of hospitalization due to SAE<br>Description: see Section 7<br>Time Frame: from the 1st study treatment in the core study up to 40 weeks post baseline visit in the core study, the patient's 2 years' corrected age and the patient's fifth birthday	See Section 9.5.2
To evaluate the visual function by assessing the visual acuity in the worse-seeing eye at the patient's fifth birthday[a]	Title: visual acuity in the worse-seeing eye at the patient's fifth birthday<br>Unit of Measure: visual acuity score (Lea symbols optotypes)<br>Description: see Section 6.4.3<br>Time Frame: at the patient's fifth birthday	See Section 9.5.1
To evaluate the absence of active ROP at 40 weeks and 52 weeks post baseline visit in the core study	Title: absence of active ROP<br>Unit of Measure: number and percentage of patients<br>Description: see Section 3.5.1<br>Timeframe: at 40 weeks and 52 weeks post baseline visit in the core study	See Section 9.5.1
To evaluate the absence of ocular structural abnormalities at or before 40 weeks post baseline visit in the core study, at the patient's 2 years corrected age and fifth birthday	Title: absence of all ocular structural abnormalities<br>Unit of Measure: number and percentage of patients<br>Description: the absence of all ocular structural abnormalities listed in Section 3.5.1 in both eyes	See Section 9.5.1

[redacted]

OBJECTIVE	Endpoint Title, Description and Reporting Time Frame for analysis and Unit of Measure	Stat Analysis Section
	Time Frame: at or before 40 weeks post baseline visit in the core study, at or before the patient's 2 years corrected age, at or before the patient's fifth birthday<br><br>Title: absence of <u>each</u> ocular structural abnormality as listed in Section 3.5.2 considered individually<br>Unit of Measure: number and percentage of patients<br>Time Frame: at or before week 40, at or before the patient's 2 years' corrected age, at or before the patient's fifth birthday	
To evaluate the recurrence of ROP up to 40 weeks and 52 weeks post baseline visit in the core study	Title: recurrence of ROP<br>Unit of Measure: number and percentage of patients with recurrence of ROP<br>Description: Recurrence of ROP is defined as ROP receiving any intervention after the 1st study treatment in the core study[d].<br>Time Frame: up to 40 weeks and 52 weeks post baseline visit in the core study	See Section 9.5.1
To assess the number of ranibizumab injections received in the treatment of patients with ROP up to and including 40 weeks post baseline visit in the core study	Title: number of ranibizumab injections<br>Unit of Measure: number of ranibizumab injections<br>Description: as per title<br>Time Frame: up to and including 40 weeks post baseline visit in the core study	See Section 9.5.2
To evaluate the refraction in each eye at the patient's 2 years' corrected age and fifth birthday[a]	Title: refraction in each eye<br>Unit of Measure: diopters<br>Description: see Section 6.4.2<br>Time Frame: at the patient's 2 years corrected age, patient's fifth birthday	See Section 9.5.1
To evaluate the physical development at the patient's 2 years' corrected age and fifth birthday[b]	Title: standing/sitting height, leg length, weight<br>Unit of Measure: centimeter, gram (as appropriate)<br>Description: see Section 6.5.2.2; leg length will be derived from standing/sitting height difference<br>Time Frame: at the patient's 2 years corrected age, at the patient's fifth birthday<br><br>Title: head circumference	See Section 9.5.2

[redacted]

OBJECTIVE	Endpoint Title, Description and Reporting Time Frame for analysis and Unit of Measure	Stat Analysis Section
	Unit of Measure: centimeter<br>Description: see Section 6.5.2.2<br>Time Frame: at the patient's 2 years corrected age	
To evaluate the health status at the patient's 2 years' corrected age and fifth birthday[b,c]	Title: respiratory function[b]<br>Unit of Measure: number and percentage of patients with oxygen supplementation; with presence of wheezing symptoms<br>Description: see Section 6.5.3.1<br><br>Title: hearing function[c]<br>Unit of Measure: number and percentage of patients with presence of hearing impairment<br>Description: see Section 6.5.3.2<br><br>Title: duration of hospitalization after first hospital discharge home; weight at first hospital discharge home<br>Unit of Measure: days/months as appropriate; gram<br>Description: see Section 6.5.3.3<br><br>Time Frame: at the patient's 2 years corrected age, at the patient's fifth birthday	See Section 9.5.2

[redacted]

OBJECTIVE	Endpoint Title, Description and Reporting Time Frame for analysis and Unit of Measure	Stat Analysis Section
[redacted]		

[redacted]

OBJECTIVE	Endpoint Title, Description and Reporting Time Frame for analysis and Unit of Measure	Stat Analysis Section
[redacted]		

[a] - Variable corresponding to "ocular effects" in the plan for specific follow-up in the Pediatric Investigation Plan (EMA)

[b] - Variable corresponding to "clinical outcomes" in the plan for specific follow-up in the Pediatric Investigation Plan (EMA)

[c] - Variable corresponding to "neurodevelopmental outcomes" in the plan for specific follow-up in the Pediatric Investigation Plan (EMA)

[d] - Supplementary laser treatments (as per CRFB002H2301 study protocol) are considered part of the complete laser treatment

[e] - At the visit as close as possible to the child's fifth birthday (see Table 6-1)

[redacted]

3 Investigational plan

3.1 Study design

This is a multicenter open-label extension study (Figure 3-1) where the VA assessment at the child's 5th birthday visit will be performed by an assessor who is masked to study treatment. Patients who have successfully completed the 24-week core study CRFB002H2301 are eligible for entry into the study (see Section 4). Visit 201/ baseline visit of the extension study can occur on the same day as the last visit in the CRFB002H2301 study.

Treatment with study ranibizumab (either as re-treatment after ranibizumab has already been injected in the same eye or as switch ranibizumab treatment from study laser therapy administered in the core study) will be permitted for eligible eyes up to and including the visit week 40 from the baseline visit in the core study (RFB002H2301) (equal to week 16 from the baseline visit in extension study, when the baseline visit in extension study coincides with the week 24 visit in the core study) [Epoch 1]. The remainder of the extension study [Epoch 2] is observational.

Assessment visits will be performed regularly throughout the Epoch 1 and 2, up to the last study visit as close as possible to the patient's fifth birthday. The assessments related to the primary objective will be performed at the patient's fifth birthday. Two interim analyses are planned, see Section 3.5. Additional interim analyses may be conducted on safety or efficacy data as required.

Figure 3-1 Study design

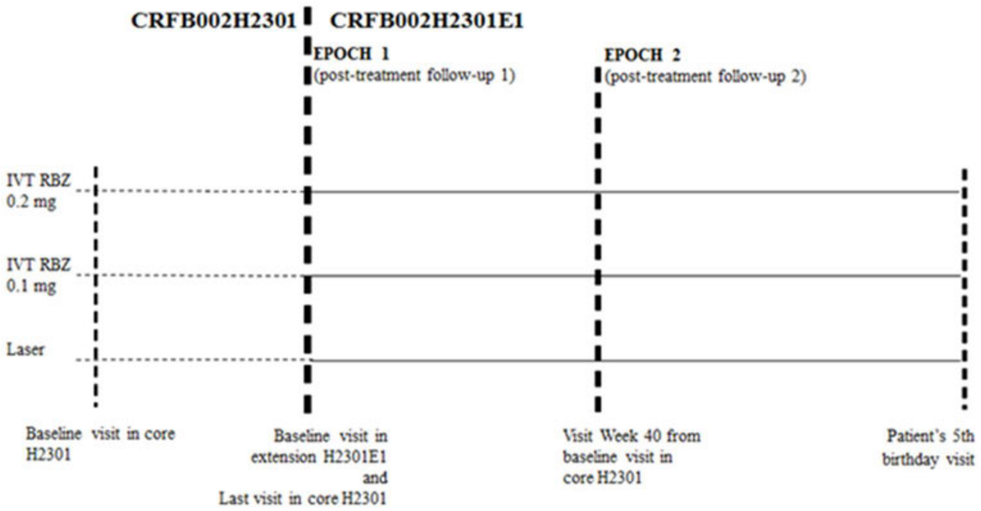

3.2 Rationale for study design

The patient population will be described in more detail in the Section 4 below.

This multicenter extension study is designed to provide long term efficacy and safety data of ranibizumab treatment compared with laser ablation therapy in ROP patients. A follow-up duration until patients are 5 years of age will also provide the opportunity for a robust

[redacted]

assessment of the visual function of the patients as well as of their physical growth and cognitive status. Late recurrence of ROP is not expected beyond 40 weeks after the first study treatment (in the core study CRFB002H2301), therefore the treatment period ends with the end of the Epoch 1. Interim analyses will be performed using a snapshot of the database available at the cut-off points. The first interim analysis will be conducted to comply with the plan for specific follow-up in the Pediatric Investigation Plan (EMA) and to support the inclusion of information related to the treatment of ROP in the Lucentis product information (in EU: Summary of Product Characteristics (SmPC)). The second interim analysis will be conducted to evaluate efficacy and safety data and report on the progress of the study to the scientific community during the course of the study. Additional interim analyses may be conducted on safety or efficacy data as required.

3.3 Rationale for dose/regimen, route of administration and duration of treatment

Treatment with study ranibizumab is permitted in the Epoch 1 for eligible eyes who have recurrence / worsening of ROP disease, in the opinion of the investigator.

The duration of the Epoch 1 (when treatment with study ranibizumab is permitted) is up to and including 40 weeks after the first study treatment in the core study CRFB002H2301. This reflects that late recurrence of ROP is not expected beyond this time point.

3.4 Rationale for choice of comparator

There is no comparator in the present extension study. Laser ablation of the peripheral avascular retina has been selected as the comparator for intravitreal ranibizumab in the core clinical study CRFB002H2301. This treatment modality is the current standard of care treatment for ROP in many countries (Royal College of Paediatrics and Child Health 2008, Hartnett and Penn 2012).

3.5 Purpose and timing of interim analyses/design adaptations

3.5.1 Interim analysis 1

The first interim analysis will be performed when the last patient has completed the last visit in core study CRFB002H2301 or when at least half of the patients enrolled in CRFB002H2301 have completed the visit corresponding to 40 weeks after the first study treatment (in the core study CRFB002H2301), whichever is the latest.

The purpose of the first interim analysis is to provide some longer term data on ocular structural abnormalities. This is to comply with the plan for specific follow-up in the Pediatric Investigation Plan (EMA). Therefore, the absence of ocular structural abnormalities will be evaluated as defined by the absence of all of the following fundus features in both eyes at or before week 40 after the first study treatment in the core study CRFB002H2301:

- Substantial temporal retinal vessel dragging causing abnormal structural features/ macular ectopia
- Retrolental membrane obscuring the view of the posterior pole
- Posterior retinal fold involving the macula
- Retinal detachment involving the macula

[redacted]

Additional objectives of the interim analysis 1 are:

- to explore the absence of active ROP in both eyes as defined by the absence of all of the following features:
  - Vessel dilatation of plus disease in at least 2 quadrants (some persisting tortuosity is allowed)
  - Extra-retinal vessels extending from the retina into the vitreous and judged to be a sign of active ROP disease
- to explore the recurrence of ROP
- to explore the number of ranibizumab injections
- to explore the safety outcomes by analyzing the type, frequency and severity of ocular and non-ocular Adverse Events

3.5.2 Interim analysis 2

The interim analysis 2 will be performed when the last patient has completed the visit which corresponds to the patient's 2 years' corrected age.

The rationale for the second interim analysis is given in Section 3.2.

The purpose of the second interim analysis is to evaluate the absence of all ocular structural abnormalities as defined in Section 3.5.1 at the patient's 2 years' corrected age.

Other objectives of the second interim analysis are:

- to evaluate the absence of each ocular structural abnormality considered individually, i.e.
  - Each ocular structural abnormality as listed above in Section 3.5.1
  - Retinal detachment not involving the macula
  - Pre-retinal fibrosis
  - Optic disc pallor
  - Optic disc swelling
  - Pigmentary disturbance in the macula
  - Atrophic changes in the macula
- [redacted]
- to evaluate the safety outcomes by analyzing the type, frequency and severity of ocular and non-ocular Adverse Events

Further objectives are to evaluate clinical and cognitive outcomes as per Table 2-1.

Additional details for the interim analyses are provided in Section 3.2 and the statistical considerations in Section 9.7.

3.6 Risks and benefits

The risk to subjects in this trial will be minimized by compliance with the eligibility criteria and study procedures and close clinical monitoring.

This study will be an extension of the core study CRF002H2301 conducted in preterm infants who received intravitreal ranibizumab injection and/or laser therapy for retinopathy of

[redacted]

prematurity. For eligible patients, the patient's parent(s) or legal guardian(s) will be invited to allow the patient to continue in the extension study. This is a vulnerable patient population due to their age and associated comorbidities. To protect their rights, safety, and well-being, only study centers and investigators experienced in managing these patients will be invited to participate in the core and extension studies.

Study ranibizumab can be administered during the Epoch 1 of the extension study to patients who, in the opinion of the investigator, have a recurrence / worsening of ROP disease. This is to ensure study treatment is still available for potential late ROP recurrences following anti-VEGF treatment (Chen 2014, Hu et al 2012). Patients not eligible for study ranibizumab treatment will receive the Standard of Care therapy as per local clinical practice. Based on published literature, late recurrences of ROP following anti-VEGF therapy are not expected beyond week 40 post-baseline treatment. The remainder of the extension study (Epoch 2) is therefore observational.

Treatment with investigational ranibizumab is associated with potential risks and benefits.

The potential benefits of intravitreal anti-VEGF in the management of ROP have been previously described (Section 1.1). Notably, a prospective randomized clinical study demonstrated that intravitreal bevacizumab, compared to laser therapy (the current Standard of Care therapy in most countries), was more efficacious in the treatment of ROP to reduce the number of recurrences and was associated with less myopia in the long term (Mintz-Hittner et al 2011, Geloneck et al 2014). Based on published information, more than 800 infants with ROP have received ranibizumab or other anti-VEGF intravitreal injections. Ranibizumab treatment has the potential advantage versus laser therapy of being able to treat ROP without causing widespread damage to the peripheral retina, thereby enabling peripheral retinal vascularization to continue in the eye, preserving the visual fields.

In adults, ranibizumab has been shown to improve vision and help prevent visual loss in adults with age related macular degeneration, visual impairment due to pathologic myopia or diabetic macular edema or retinal vein occlusion.

Potential risks with the use of intravitreal anti-VEGF agents, as identified in published case reports, include the development of late retinal detachment and reduction in systemic VEGF levels (Honda et al 2008, Jang et al 2010, Lee et al 2010, Zepeda-Romero et al 2010, Hu et al 2012, Sato et al 2012, Hoerster et al 2013). Furthermore, risks associated with the intravitreal injection procedure include endophthalmitis, retinal detachment, traumatic cataract, and increased intraocular pressure.

Serum VEGF levels in ROP infants following ranibizumab have been measured in two studies (Hoerster et al. 2013, Zhou et al. 2015). Hoerster et al. 2013 described one infant whose VEGF levels were reduced 1 week after ranibizumab injection, were further reduced below detection limit at week 3 and returned to normal levels at week 4. Zhou et al. 2015 measured plasma VEGF levels before and 1 day after ranibizumab injection in 5 ROP infants: plasma VEGF levels were reduced to between 9% and 37% of pre-dose VEGF levels 1 day after the injection and normalized after 1 week.

Potential risks of systemic inhibition of VEGF may include disturbances of organ and neurological development, renal impairment, bone abnormalities, and thromboembolic events.

[redacted]

These effects were observed in studies using animal knockout models, in nonclinical and clinical studies with high systemic doses of anti-VEGF agents or in clinical studies of intravitreal anti-VEGF in adults with predisposing comorbidities. No such systemic adverse effects have been reported in the published reports of intravitreal anti-VEGF therapy in ROP. Nonetheless, all patients will be monitored during the study for any new onset AEs.

As with all therapeutic proteins, there is a potential for immunogenicity with ranibizumab. In adult patients, the pretreatment incidence of immunoreactivity to ranibizumab was 0% to 5%. After monthly dosing with ranibizumab for 6 to 24 months, antibodies to ranibizumab were detected in approximately 1% to 9% of patients. The clinical significance of immunoreactivity to ranibizumab is unclear. Among neovascular age-related macular degeneration patients with the highest levels of immunoreactivity, some were noted to have iritis or vitritis. Intraocular inflammation was not observed in the retinal vein occlusion or diabetic macular edema patients with the highest levels of immunoreactivity. The number of administrations of ranibizumab in this study is less than that for the treatment of adult conditions. In addition, neonates have a less developed adaptive immune response (Adkins et al 2004). Hence, the potential risk of immunogenicity in this study population is likely to be low.

An independent Data Monitoring Committee (DMC) was established to monitor the safety of the trial participants in the core study and will continue evaluations in this extension study until the last patient has completed visit 301 (i.e. week 52 after the 1st study treatment in the core study) or discontinued the study (see Section 8.4), to ensure that the trial is being conducted with the highest scientific and ethical standards, and make appropriate recommendations based on the data seen.

Epoch 2 is observational. Patients will receive standard of care therapy as needed and as per local clinical practice and will be followed-up to monitor long term efficacy and safety outcomes of ranibizumab treatment as compared to laser ablation therapy. This will address an unmet need of the medical community to understand the long term benefits and risks of ranibizumab treatment compared to laser therapy in the treatment of ROP.

4 Population

The study population will consist of male and female preterm infants/children who have successfully completed the CRFB002H2301 study.

4.1 Inclusion criteria

Patients eligible for inclusion in this study have to fulfill all of the following criteria at baseline of the extension study prior to receiving the first study related procedure (including investigational treatment):

1. Signed informed consent from parent(s) or legal guardian(s), in compliance with local requirements
2. The patient successfully completed the core study CRFB002H2301, as defined by providing assessments at the Visit 112 or, if appropriate, at the last of the additional assessment visits as per protocol in CRFB002H2301, whichever is latest
3. The patient received study treatment in both eyes at baseline of study CRFB002H2301

[redacted]

4.2 Exclusion criteria

Patients fulfilling any of the following criteria at study entry are not eligible for inclusion in this study. No additional exclusions may be applied by the investigator, in order to ensure that the study population will be representative of all eligible patients.

1. Patient has a medical condition or personal circumstance which precludes study participation or compliance with study procedures, as assessed by the Investigator
2. Patient has been discontinued from the core study CRFB002H2301 at any time

5 Treatment

5.1 Study treatment

5.1.1 Investigational and control drugs

5.1.1.1 Epoch 1

- 0.1 mg and 0.2 mg ranibizumab

Ranibizumab solution for injection will be supplied (open label) in vials. Each vial contains ranibizumab in the concentration of 10 mg/mL (RFB002 0.5 mg/0.05 mL). Ranibizumab is formulated as a sterile solution aseptically filled in a sterile glass vial for single use only. The content of the vial must not be split. The vials will be supplied by Novartis Drug Supply Management.

Ranibizumab must be stored according to the label instructions and it must be kept in a secure locked facility.

Each vial will be labeled with the appropriate information. Medication labels will comply with the legal requirements and will be printed in the local language. The storage conditions for study drug will be described on the medication label.

Novartis will provide sufficient supplies of ranibizumab to allow for completion of Epoch 1.

Treatment administration is detailed in Section 5.5.4 of this protocol.

5.1.1.2 Epoch 2

There is no investigational/ control drug in Epoch 2.

5.1.2 Additional treatment

No additional treatment is included in this trial.

5.2 Treatment arms

Treatment arm assignment will remain the same as in the core study.

[redacted]

5.3 Treatment assignment and randomization

At visit 201 (extension baseline), the investigator or his/her delegate will contact the IRT after confirming that the patient fulfills all the inclusion/exclusion criteria. Treatment arm assignment and patient identifier will remain the same as in the core study.

During Epoch 1, the investigator or his/her delegate will contact the IRT upon decision to administer study treatment. IRT will specify a unique medication number for the package of study drug to be dispensed to the patient.

The investigator must contact the IRT to register the patient's completion of Epoch 1.

5.4 Treatment blinding

This is an open-label study where the VA assessment at the child's 5th birthday visit will be performed by an assessor who is masked to study treatment. The investigators, patients/parents/legal guardians and the Clinical Trial Team (CTT) are unmasked to the treatments administered during the core and extension studies. The measures defined in the core study CRFB002H2301 to minimize bias related to treatment knowledge will remain implemented by the CTT until the core study clinical database is locked. They will be provided in the Data Management Plan.

5.5 Treating the patient

5.5.1 Patient numbering

Each patient is uniquely identified in the study by a combination of his/her center number and patient number. The patient identifier will remain the same in the extension study as in the core study CRFB002H2301. The investigator should contact the Novartis medical monitor in case of special circumstances (e.g. site transfer).

The investigator or his/her staff will contact the IRT and provide the requested identifying information for the patient to register them into the IRT.

5.5.2 Dispensing the study drug

Each study site will be supplied by Novartis with (open-label) study drug.

The study drug packaging has a 2-part label. A unique medication number (kit number) is printed on each part of this label. Investigator staff will identify the study drug package(s) to dispense to the patient by contacting the IRT and obtaining the medication number(s).

Immediately before dispensing the package to the patient, investigator staff will detach the tear-off label from the packaging and affix it to the source document (Drug Label Form) for that patient's unique subject number.

5.5.3 Handling of study and additional treatment

5.5.3.1 Handling of study treatment

Study treatment (ranibizumab) must be received by a designated person at the study site, handled and stored safely and properly, and kept in a secured location to which only the

[redacted]

investigator and designees have access. Upon receipt, all study treatment must be stored according to the instructions specified on the labels. Clinical supplies are to be dispensed only in accordance with the protocol. Technical complaints are to be reported to the respective Novartis CPO Quality Assurance.

Medication labels will be in the local language and comply with the legal requirements of each country. They will include storage conditions for the study treatment but no information about the patient except for the medication number.

The investigator must maintain an accurate record of the shipment and dispensing of study treatment in a drug accountability log. Monitoring of drug accountability will be performed by monitors during site visits or remotely and at the completion of the trial.

At the conclusion of the study, and as appropriate during the course of the study (e.g. after completion of Epoch 1), the investigator will return all unused study treatment, packaging, drug labels, and a copy of the completed drug accountability log to the Novartis monitor or to the Novartis address provided in the investigator folder at each site.

5.5.3.2 Handling of additional treatment

Not applicable.

5.5.4 Instructions for prescribing and taking study treatment

0.1 mg and 0.2 mg ranibizumab

On day(s) when study treatment is administered, all assessments must be conducted before administration except when specified otherwise in Section 6.

All study treatment administration during the study must be recorded in the Dose Administration Record eCRF.

Instructions on preparation of the aseptic field, preparation of the ranibizumab for injection, the intravitreal injection technique to minimize the risk of complications in patients, and information on pre- and post-injection procedures are provided in the study operation manual.

Treatment with ranibizumab (either as re-treatment after ranibizumab has already been injected in the same eye or as switch ranibizumab treatment from study laser therapy administered in the core study) will be permitted for eligible eyes up to and including the study visit occurring 40 weeks after the first study treatment in the core study CRFB002H2301 (Epoch 1). The following rules apply:

- Treatment with investigational ranibizumab will only be permitted for recurrence / worsening of ROP, as judged by the investigator. Details (e.g. reason) must be documented in the CRF.
- No further investigational ranibizumab treatment can be administered to the eye after it was switched to study laser therapy (in the core study CRFB002H2301). If the other eye was not switched to study laser therapy (in the core study CRFB002H2301) then this eye could be administered ranibizumab re-treatment.

[redacted]

- A maximum of 3 study ranibizumab treatments can be administered to each eye throughout the core and extension studies (i.e. including injections received in the core study CRFB002H2301 and extension study CRFB002H2301**E1**).
- The minimum time interval between ranibizumab injections in the same eye is 28 days.
- Only the eye which needs treatment will be treated. If both eyes need treatment, then both eyes will be treated.
- Investigational ranibizumab is not to be administered to an eye that has developed stage 4 or 5 ROP. The development of any complications of ROP, which is assessed by the Investigator as not suitable for treatment with ranibizumab, should be managed as appropriate and the treatment given documented in the eCRF.
- Investigational ranibizumab treatment must be discontinued for a patient under circumstances listed in Table 5-2.
- No further investigational ranibizumab treatment can be administered if the patient was discontinued from study treatment in the core study CRFB002H2301.

The dose of study ranibizumab to be administered (if the above rules apply) will be as follows:

Table 5-1 Study ranibizumab dose in Extension study based on treatment received at the baseline visit in core study (CRFB002H2301)

Treatment received at the baseline visit in Core study (CRFB002H2301)	Ranibizumab dose in Extension study (CRFB002H2301**E1**)
RBZ 0.1mg	RBZ 0.1mg
RBZ 0.2mg	RBZ 0.2mg
Laser	RBZ 0.2mg

After treatment with investigational ranibizumab, the visits as per Additional Assessment Schedule in Table 6-2 will be performed. All study ranibizumab treatments must be recorded in the IRT system.

Patients with recurrence / worsening of ROP, in the opinion of the investigator, who are not eligible or suitable for treatment with ranibizumab should be treated with Standard of Care therapy as per local clinical practice. Details of any treatments or procedures should be documented in the CRF.

5.5.5 Permitted dose adjustments and interruptions of study treatment

Investigational treatment dose adjustments and/or interruptions are not permitted.

5.5.6 Rescue medication

Patients with recurrence/worsening of ROP (as judged by the investigator) who are not eligible or suitable for treatment with ranibizumab as per Section 5.5.4 should be treated with Standard of Care therapy, considering instructions for study treatment discontinuation in Table 5-2. Details of any treatments or procedures must be recorded in the Concomitant medications or Surgical and medical procedures CRF as applicable.

[redacted]

5.5.7 Concomitant medication

The investigator must instruct the patient's parent(s) or legal guardian(s) to notify the study site about any new medications the patient takes since the patient completed the core study and throughout the extension study. All medications and procedures (including physical therapy and blood transfusions) administered when the patient was enrolled into the extension study and after must be recorded in the prior and concomitant medications eCRF or the surgical and medical procedures eCRF as appropriate.

Concomitant medications which are ongoing at the time of the last visit in the core study CRFB002H2301 must be recorded, and entries in the extension study database should match those in the core study database as appropriate.

For patients whose baseline visit of the extension study does not occur on the same day as the last visit in the CRFB002H2301 core study, relevant medications and procedures (e.g. related to ocular treatment) which started after the last visit in the core study and before the baseline visit in the extension study will also be recorded.

Each concomitant drug and procedures must be individually assessed against all treatments in Table 5-2. If in doubt the investigator should contact the Novartis medical monitor before allowing a new medication to be started.

From Visit 301, concomitant medications/ non-drug therapies related to common childhood infections and accidents/injuries if they are not SAEs (Section 7.1) are not required to be reported in the CRF.

5.5.8 Prohibited medication

There is no prohibited medication in this study, however, the use of the treatments in Table 5-2 will prohibit further use of investigational ranibizumab. These rules apply from the last visit in the core study.

Table 5-2 Treatment which prohibits further use of study ranibizumab

Medication	Action to be taken
Any intravitreal anti-VEGF agent other than the investigational drug	Discontinue study treatment
Any systemic anti-VEGF agent	Discontinue study treatment
Any other nonsurgical treatment of ROP	Discontinue study treatment
Any surgical treatment of ROP (e.g. cryotherapy, vitrectomy, laser)	Discontinue study treatment
Any other investigational medicinal product as part of another clinical study, except vitamins and minerals	Discontinue study treatment

Abbreviations: ROP – retinopathy of prematurity; VEGF – vascular endothelial growth factor

5.5.9 Emergency breaking of assigned treatment code

Not applicable. This is an open-label study.

[redacted]

5.6 Study Completion and Discontinuation

5.6.1 Study completion and post-study treatment

A patient will be considered to have completed the study when the patient has completed the last visit planned in the protocol.

Continuing care according to Standard of Care therapy as per local clinical practice should be provided by investigator and/or referring physician based on patient availability for follow-up.

5.6.2 Discontinuation of Study Treatment

Discontinuation of study treatment for a patient occurs when study drug is stopped earlier than the protocol planned duration (i.e. during Epoch 1), and can be initiated by either the patient's parent(s) or legal guardian(s) or the investigator.

The investigator must discontinue study treatment for a given patient if, on balance, he/she believes that continuation would negatively impact the risk/benefit of trial participation.

Study treatment must be discontinued for a patient under the following circumstances:

- Patient's parent(s) or legal guardian(s) wish
- Use of treatment as per Table 5-2
- Any situation in which study treatment administration might result in a safety risk to the patient

If discontinuation of study treatment occurs, the patient should NOT be considered withdrawn from the study. The patient should return for assessments as described in Table 6-1. The investigator must determine the primary reason for the patient's premature discontinuation of study treatment and record this information on the Dosage Administration eCRF.

After study treatment discontinuation, all data should be collected as per Table 6-1 and, at a minimum, in abbreviated visits, the following data should be collected at clinic visits or via telephone visits according to the study visit schedule:

- new / concomitant treatments
- Adverse Events/Serious Adverse Events

5.6.3 Premature patient withdrawal from the study

The patient must be withdrawn from the study if, on balance, the investigator believes that continuation would negatively impact the risk/benefit of the patient's participation in the trial. A final evaluation at the time of the patient's study withdrawal should be made as detailed in Table 6-1. The investigator must also contact the IRT to register the patient's withdrawal from the study if it occurs before the patient has completed the last visit in Epoch 1 and document the decision to withdraw the patient in the Study Completion CRF and patient's source documents.

[redacted]

5.6.3.1 Withdrawal of informed consent

Patient’s parent(s) or legal guardian(s) may voluntarily withdraw consent for the patient to participate in the study for any reason at any time. Withdrawal of consent occurs only when patient’s parent(s) or legal guardian(s):

- Does not want the patient to participate in the study anymore and

Does not allow further collection of personal data In this situation, the investigator should make a reasonable effort (e.g. telephone, e-mail, letter) to understand the primary reason for the patient's parent(s) or legal guardian decision to withdraw his/ her consent to the patient's participation and record this information.

Study treatment must be discontinued and no further assessments conducted, and the data that would have been collected at subsequent visits will be considered missing.

Further attempts to contact the patient’s parent(s) or legal guardian(s) are not allowed unless safety findings require communicating or follow-up.

A final evaluation at the time of the patient’s study withdrawal should be made as detailed in Table 6-1.

Novartis will continue to keep and use collected study information according to applicable law.

The investigator must also contact the IRT to register the patient’s withdrawal from the study if it occurs before the patient has completed the last visit in Epoch 1 and document the patient's parent(s) or legal guardian(s) decision to withdraw consent in the Study Completion CRF and patient's source documents.

5.6.4 Loss to follow-up

For patients whose status is unclear because they fail to appear for study visits and their parent(s) or legal guardian(s) have not stated an intention to discontinue or withdraw their child, the investigator should show "due diligence" by documenting in the source documents steps taken to contact the patient’s parent(s) or legal guardian(s), e.g. dates of telephone calls, registered letters, etc.

5.6.5 Early study termination by the sponsor

The study can be terminated by Novartis at any time for any reason. This may include reasons related to the benefit risk assessment of participating in the study, practical reasons, or for regulatory or medical reasons (including slow enrolment). Should this be necessary, the patient must be seen as soon as possible and treated as a prematurely withdrawn patient. The investigator may be informed of additional procedures to be followed in order to ensure that adequate consideration is given to the protection of the patient’s interests. The investigator will be responsible for informing the Institutional Review Board/Independent Ethics Committee (IRBs/IECs) of the early termination of the trial.

6 Visit schedule and assessments

Table 6-1 and Table 6-2 list all study related assessments and indicate with an “x” when the visits are performed. When a visit as per the additional assessment schedule coincides with a

[redacted]

visit as per the main assessment schedule, assessments have to be performed as per the main assessment schedule. Non study-related assessments and follow-ups which are performed according to local medical practice should not be considered as unscheduled study visits.

Patients/subjects should be seen for all visits on the designated day, or as close to it as possible (visit window as indicated on the relevant assessment schedule tables should be considered). Alternatively, Visit 304 may be conducted remotely by phone, at the investigator's discretion. Study visit assessments should be carried out on the same day. If required, assessments may be conducted on two consecutive days, in order to decrease patient burden. Missed visits should be re-scheduled as soon as possible. Missed or rescheduled visits should not lead to automatic discontinuation. Patients/subjects who prematurely discontinue the study for any reason should be scheduled for a visit as soon as possible, at which time the assessments listed for Visit 399 (final visit) will be performed unless otherwise specified.

Patient's parent(s) or legal guardian(s) will be contacted for safety evaluations during the 30 days following the last study visit (see Section 7.2).

[redacted]

Table 6-1 Main Assessment Schedule

	Epoch 1 (Post-treatment follow-up 1)			Epoch 2 (Post-treatment follow-up 2)					
**Visit**	**201[a] (baseline)**	**202**	**203**	**301**	**302**	**303**	**304**	**399 incl. premature withdrawal**	**Supplemental visit [n]**
**Timepoint (referring to the Day 1 in the study CRFB002H2301)**	Day of the patient's last visit in core CRFB002H2301[a]	Week 32 (+/- 1 week)	Week 40 (+/- 1 week)	Week 52 (+/- 2 weeks)	Child's 2 years' corrected age[i] (+/- 4 weeks)	Child's 3 years' corrected age[i] (+/- 4 weeks)	Child's 4th birthday[j] (+/- 4 weeks)	Child's 5th birthday[j] (+/- 12 weeks)	
Obtain informed consent	X								
Inclusion/Exclusion	X								
Demography	X								
Relevant medical conditions	X[c]								
Adverse events	X[b]	X	X	X	X	X	X	X	X
Concomitant medication use	X[d]	X	X	X	X	X	X	X	X
Health status (respiratory and hearing function)					X			X	
Hospital discharge[h]	X	X	X	X	X	X	X	X	X
Blood pressure					X				
Height, weight					X			X	
Head circumference					X				
Laboratory tests					X				

[redacted]

	**Epoch 1 (Post-treatment follow-up 1)**			**Epoch 2 (Post-treatment follow-up 2)**					
**Visit**	**201[a] (baseline)**	**202**	**203**	**301**	**302**	**303**	**304**	**399 incl. premature withdrawal**	**Supplemental visit [n]**
**Timepoint (referring to the Day 1 in the study CRFB002H2301)**	Day of the patient's last visit in core CRFB002H2301[a]	Week 32 (+/- 1 week)	Week 40 (+/- 1 week)	Week 52 (+/- 2 weeks)	Child's 2 years' corrected age[i] (+/- 4 weeks)	Child's 3 years' corrected age[i] (+/- 4 weeks)	Child's 4th birthday[j] (+/- 4 weeks)	Child's 5th birthday[j] (+/- 12 weeks)	
Ocular examination[e]		X	X	X	X			X	
Fundus examination by ophthalmoscopy		X	X	X	X			X	
[redacted]									
Ocular and visual function					X[m]	X[m]		X[g,q]	X[o]
[redacted]									
Epoch 1 disposition[f]			X						
Study disposition								X	

a – Visit 201 (baseline) of CRFB002H2301**E1** can occur on the same day as the patient's last visit in CRFB002H2301 (i.e. on Day 169 / Visit 112 of CRFB002H2301 or at the last of the additional assessments visit as per protocol in CRFB002H2301). b - Adverse Events which are ongoing at the patient's last visit in CRF002H2301 and at the baseline visit in the extension study must be recorded and entries in the extension study database should match those in the core study database as appropriate.

[redacted]

	Epoch 1 (Post-treatment follow-up 1)			Epoch 2 (Post-treatment follow-up 2)					
**Visit**	**201[a] (baseline)**	**202**	**203**	**301**	**302**	**303**	**304**	**399 incl. premature withdrawal**	**Supplemental visit [n]**
**Timepoint (referring to the Day 1 in the study CRFB002H2301)**	Day of the patient's last visit in core CRFB002H2301[a]	Week 32 (+/- 1 week)	Week 40 (+/- 1 week)	Week 52 (+/- 2 weeks)	Child's 2 years' corrected age[i] (+/- 4 weeks)	Child's 3 years' corrected age[i] (+/- 4 weeks)	Child's 4th birthday[j] (+/- 4 weeks)	Child's 5th birthday[j] (+/- 12 weeks)	

c - When baseline visit of the extension study does not occur on the same day as the last visit in the core study, relevant medical conditions which started and/or ended after the last visit in the core and before the baseline visit in the extension study will be recorded (see Section 6.2).

d – Concomitant medications which are ongoing at the patient's last visit in CRF002H2301 must be recorded and entries in the extension study database should match those in the core study database as appropriate. For patients whose baseline visit of the extension study does not occur on the same day as the last visit in the CRFB002H2301 core study, relevant medications and procedures which started after the last visit in the core study and before the baseline visit in the extension study will also be recorded (see Section 5.5.7).

e - Including examination of the anterior segment and lens

f - To be recorded once upon Epoch 1 completion

g - Will not be performed in case of patient's premature withdrawal

h - To be recorded once upon first hospital discharge home

i – Visit should be scheduled as close as possible when child reaches this corrected age

j – Visit should be scheduled as close as possible to the child's appropriate birthday

[redacted]

m - Assessment of presence/ absence of [redacted] refraction. See Section 6.4.2.

n – The supplemental visit should take place as early as possible after the protocol amendment is implemented. If the date of this visit is within 2 months prior the next scheduled visit, then this supplemental visit may be delayed and performed with the next scheduled visit

[redacted]

p – ETDRS visual acuity test (using Lea symbols) to be performed by a masked VA assessor: See Section 6.4.3.

q – Includes assessments (m) and vision function form (peripheral vision).

[redacted]

Table 6-2 Additional Assessment Schedule – Treatment with Ranibizumab

Visit	29y1[f]	29y2[f]	29y3[f]	29y4[f]
Days after ranibizumab treatment[a] (visit window)	0	3 (-2 day/+1 day)	7 (±2 day)	14[b] (±4 days)
Adverse events	X	X	X	X
Concomitant medication use /Surgical and medical procedures	X	X	X	X
Ocular examination[c]	X[d]	X	X	X
Fundus examination	X[d]	X	X	X
Administer ranibizumab[e]	X			

a – Up to 2 re-treatments (including treatments administered in the Core Study) are allowed for each eye if required

b – After this visit, the patient resumes the next visit according to their original Main Assessment Schedule

c – Includes examination of the anterior chamber and lens

d – Assessments to be conducted before administration of ranibizumab treatment

e – The ranibizumab treatment dose will be as per Table 5-1

f – y is the number corresponding to the ranking of the post-baseline treatment, continuing from the core (e.g. 1 corresponds to the 1st treatment after the core study baseline treatment)

[redacted]

6.1 Information to be collected on screening failures

Not applicable. There is no screening Epoch.

6.2 Patient demographics/other baseline characteristics

Patient demographic and baseline characteristic data to be collected on all patients include: race and ethnicity (based on that of the mother), start date of the mother's last normal menstrual period, sex, year of birth, gestational age at birth (by completed weeks). Entries in the extension study database should match those in the core study database.

For patients whose baseline visit of the extension study does not occur on the same day as the last visit in the CRFB002H2301 core study, relevant medical condition which started and/or ended after the last visit in the core study and before the baseline visit in the extension study will be recorded in the extension study database.

6.3 Treatment exposure and compliance

Every time that ranibizumab treatment is to be administered, IRT needs to be contacted for the medication (kit) number. Information regarding investigational treatment administration will be collected in the Dose Administration Record in the eCRF.

Exposure to ranibizumab treatment will be based on the number of injections by eye and overall (as databased on IRT as well as on the Drug Administration Record eCRF). Compliance with ranibizumab treatment will be assessed by the field monitor at each visit until completion of Epoch 1 using vial counts and information provided by the pharmacist or by the investigator.

6.4 Efficacy

6.4.1 Ophthalmic fundus features

The presence of the following fundus features will be assessed by indirect ophthalmoscopy in both eyes by the investigator at study visits specified in Table 6-1 and Table 6-2:

- Features of active ROP disease:
    - ROP disease – zone, stage, extent by clock hours, and characteristics of vascular changes of plus disease (extent by quadrants and severity) (up to Visit 301 included)
    - Extra-retinal vessels extending from the retina into the vitreous and judged to be a sign of active ROP disease (up to Visit 301 included)
- Features of late sequelae of ROP /ocular structural abnormalities:
    - Retrolental membrane obscuring the view of the posterior pole
    - Substantial temporal retinal vessel dragging causing abnormal structural features
    - Posterior retinal fold involving the macula
    - Retinal detachment involving the macula
    - Retinal detachment not involving the macula
    - Pre-retinal fibrosis
    - Optic disc pallor (from Visit 301 included onwards)

[redacted]

- Optic disc swelling (from Visit 301 included onwards)
- Pigmentary disturbance in the macula (from Visit 301 included onwards)
- Atrophic changes in macula (from Visit 301 included onwards)

[redacted]

6.4.2 Ocular and visual function

The following will be assessed in both eyes by the investigator:

[redacted]

- Refraction (in spherical equivalence) as assessed by retinoscopy or auto-refraction after cycloplegia

[redacted]

Visual acuity assessments will be performed as described in Section 6.4.3.

Additional information on the conduct of these assessments will be described in the study operation manual.

6.4.3 Visual Acuity (VA)

Visual acuity assessments (ETDRS) at the child's 5th birthday visit must be performed by a masked assessor who does not know about the study treatment the patient received at baseline of the core study CRFB002H2301.

Detailed instructions on how to perform visual acuity assessments are provided in the study operations manual.

[redacted]

6.4.3.2 ETDRS visual acuity test

Visual acuity will be tested in each eye at the child's 5th birthday visit by a masked assessor. VA measurements will be taken in a sitting position at an initial test distance of 3 meters using Lea Symbols charts. VA will be tested using the child's current refractive index, e.g. child wearing prescription glasses/ contact lenses as applicable.

[redacted]

The VA assessment must be performed prior to any other assessments unless specified otherwise (see Section 5.5.4).

[redacted]

6.4.4 Appropriateness of efficacy assessments

The fundus features assessed in this study correspond with morphologic evidence of persistence or complications resulting from ROP, and are associated with poor visual outcome. Similar measures have been used in previous studies assessing treatments for ROP (Cryotherapy for Retinopathy of Prematurity Cooperative Group 1988, Good 2004, Mintz-Hittner et al 2011).

Ocular and visual function assessments and VA assessment are standard visual function assessments (Kvarnström 2005, Becker 2002).

6.5 Safety

6.5.1 Ocular examination

The presence of fundus features abnormalities will be assessed in both eyes at study visits described in Section 6.4.1).

Ocular examination of structures in addition to that of the fundus will be performed in both eyes, including assessment of anterior ocular structures and the lens, described in Table 6-1.

Clinically significant abnormal findings will be reported as AEs in the CRF except for specific events as defined in Section 7.1.

6.5.2 Vital signs

6.5.2.1 Blood pressure

No Blood Pressure measurement is required to be performed by the protocol. Instead, the most recent systolic and diastolic blood pressure [mm Hg] measured as part of the routine clinical care should be recorded in the eCRF at visits specified in Table 6-1.

Information must be included in the source documentation at the study site. Significant findings which meet the definition of an Adverse Event must be recorded on the Adverse Event CRF.

6.5.2.2 Standing height, sitting height, weight and head circumference

For the assessment of standing/sitting heights, weight and head circumference, the patient should be wearing minimal clothing (e.g., a clean diaper if applicable and undershirt). Study staff trained in the assessment of infants or children will perform these assessments as applicable.

Standing and sitting height in centimeters will be measured using a stadiometer or according to local clinical practice. Leg length will be derived from the difference between standing height and sitting height.

Body weight will be measured using an electronic scale or according to local clinical practice.

The maximum occipitofrontal head circumference in centimeters will be measured using a tape measure.

[redacted]

6.5.3 Health status

6.5.3.1 Respiratory function status

Patients' respiratory function status will be assessed at visits specified in Table 6-1 by recording the use of any oxygen supplementation and the presence, frequency and severity of wheezing symptoms in the last 12 months.

6.5.3.2 Hearing function status

Patient's hearing function status assessment will include the presence of hearing impairment and the use of hearing aids. Usual hearing (normal or near normal; hearing loss corrected with aids (moderate or severe); hearing loss not corrected with aids (profound); no useful hearing even with aids) will be assessed.

6.5.3.3 Hospitalization

Duration of hospitalization (from birth to first hospital discharge home) and weight at the first discharge from hospital home will be collected, only if not already recorded during the core study.

6.5.4 Laboratory evaluations

No laboratory evaluations are required per protocol (e.g., hematology, clinical chemistry or urinalysis). However, data from tests which were performed as part of routine clinical practice will be collected. At visits specified in Table 6-1, the most recent laboratory values for the tests below will be collected on the eCRF, where such data are available.

6.5.4.1 Hematology

Hematology will include hemoglobin (Hb), white cell count (WCC) and platelet count (PLT).

6.5.4.2 Clinical chemistry

Serum chemistry data will include urea and electrolytes (sodium, potassium, creatinine and urea/blood urea nitrogen (BUN)) and liver function tests (bilirubin, albumin, total protein, aspartate transaminase (AST), alanine aminotransferase (ALT), alkaline phosphatase (ALP)).

6.5.4.3 Urinalysis

Urinalysis will include urine protein.

6.5.5 Appropriateness of safety measurements

The safety assessments selected are standard for this patient population.

[redacted]

[redacted]

7 Safety monitoring

7.1 Adverse events

An adverse event (AE) is any untoward medical occurrence (e.g., any unfavorable and unintended sign [including abnormal laboratory findings], symptom or disease) in a subject or clinical investigation subject *after parent(s)/ legal guardian(s) provided written informed consent* for their child's participation in the study until the end of study visit. Therefore, an AE

[redacted]

may or may not be temporally or causally associated with the use of a medicinal (investigational) product.

In this study, the following safety events will be collected as study endpoints only and are not to be additionally recorded as an AE or SAE. These specific events are captured on the endpoint page of the eCRF only, and investigators must not report these on an AE/SAE page of the eCRF:

- Substantial temporal retinal vessel dragging causing abnormal structural features/ macular ectopia
- Retrolental membrane obscuring the view of the posterior pole
- Posterior retinal fold involving the macula
- Retinal detachment
- Pre-retinal fibrosis
- Optic disc pallor (from Visit 301 included onwards)
- Optic disc swelling (from Visit 301 included onwards)
- Pigmentary disturbance in the macula (from Visit 301 included onwards)
- Atrophic changes in macula (from Visit 301 included onwards)

In this study, from Visit 301, common childhood infections and accidents/ injuries are not required to be reported as AEs, unless the AE meets one of the serious criteria (SAEs) (Section 7.2.1).

All other AEs and SAEs, whether or not considered related to ranibizumab by the investigator, must be reported.

The occurrence of adverse events must be sought by non-directive questioning of the patient's parent(s) or legal guardian(s) at each visit during the study. Adverse events also may be detected when they are volunteered by the patient's parent(s) or legal guardian(s) during or between visits or through physical examination findings, laboratory test findings, or other assessments.

Abnormal laboratory values or test results constitute adverse events only if they fulfill at least one of the following criteria:

- they induce clinical signs or symptoms,
- they are considered clinically significant,
- they require therapy

Clinically significant abnormal laboratory values or test results must be identified through a review of values outside of normal ranges/clinically notable ranges, significant changes from baseline or the previous visit, or values which are considered to be non-typical in patient with underlying disease. Investigators have the responsibility for managing the safety of individual patient and identifying adverse events.

Adverse events must be recorded in the Adverse Events CRF under the signs, symptoms or diagnosis associated with them, accompanied by the following information:

- Severity grade
  - mild: usually transient in nature and generally not interfering with normal activities
  - moderate: sufficiently discomforting to interfere with normal activities

[redacted]

    - severe: prevents normal activities
- Site (non-ocular/ocular) and laterality (bilateral/left eye/right eye), if an ocular AE
- Relationship to the study treatment and to the study procedure (e.g. intravitreal injection)
- Duration (start and end dates) or if the event is ongoing an outcome of not recovered/not resolved or recovering/resolving must be reported
- Whether it constitutes a serious adverse event (SAE - see Section 7.2 for definition of SAE)
- If it constitutes an SAE (see Section 7.2 for definition of SAE), seriousness criteria (e.g. death, requires or prolongs hospitalization)
- Action taken with investigational treatment. All adverse events must be treated appropriately. Treatment may include one or more of the following: no action taken (e.g., further observation only)/temporarily interrupted/permanently discontinued due to this adverse event/unknown/not applicable
- Medications or therapies taken (no concomitant medication or non-drug therapy/ concomitant medication or non-drug therapy)
- Outcome (not recovered/not resolved; recovered/resolved; recovering/resolving, recovered/resolved with sequelae; fatal; or unknown)

Once an adverse event is detected, it must be followed until its resolution or until it is judged to be permanent, and assessment must be made at each visit (or more frequently, if necessary) of any changes in severity, the suspected relationship to the study drug, the interventions required to treat it, and the outcome.

Information about common side effects already known about the investigational drug can be found in the Investigator Brochure (IB). This information will be included in the informed consent and should be discussed with the patient's parent(s) or legal guardian(s) during the study as needed. Any new information regarding the safety profile of the medicinal product that is identified between IB updates will be communicated as appropriate, for example, via an Investigator Notification or an Aggregate Safety Finding. New information might require an update to the informed consent and has then to be discussed with the patient's parent(s) or legal guardian(s).

The investigator must also instruct each patient's parent(s) or legal guardian(s) to report any new adverse event (beyond the protocol observation period) that the patient's parent(s) or legal guardian(s), or the patient's personal physician, believes might reasonably be related to study treatment. This information must be recorded in the investigator's source documents; however, if the AE meets the criteria of an SAE, it must be reported to Novartis.

7.2 Serious adverse events

7.2.1 Definition of SAE

An SAE is defined as any adverse event [appearance of (or worsening of any pre-existing)] undesirable sign(s), symptom(s) or medical conditions(s)) which meets any one of the following criteria:

- is fatal or life-threatening

- results in persistent or significant disability/incapacity
- constitutes a congenital anomaly/birth defect
- requires inpatient hospitalization or prolongation of existing hospitalization, unless hospitalization is for:
  - routine treatment or monitoring of the studied indication, not associated with any deterioration in condition (specify what this includes)
  - elective or pre-planned treatment for a pre-existing condition that is unrelated to the indication under study and has not worsened since signing the informed consent
  - treatment on an emergency outpatient basis for an event not fulfilling any of the definitions of a SAE given above and not resulting in hospital admission
  - social reasons and respite care in the absence of any deterioration in the patient's general condition
- is medically significant, e.g. defined as an event that jeopardizes the patient or may require medical or surgical intervention.

All malignant neoplasms will be assessed as serious under "medically significant" if other seriousness criteria are not met.

Life-threatening in the context of an SAE refers to a reaction in which the patient was at risk of death at the time of the reaction; it does not refer to a reaction that hypothetically might have caused death if it were more severe (see Annex IV, ICH-E2D Guideline).

Medical and scientific judgment should be exercised in deciding whether other situations should be considered serious reactions, such as important medical events that might not be immediately life threatening or result in death or hospitalization but might jeopardize the patient or might require intervention to prevent one of the other outcomes listed above. Examples of such events are intensive treatment in an emergency room or at home for allergic bronchospasm, blood dyscrasias or convulsions that do not result in hospitalization or development of dependency or abuse (see Annex IV, ICH-E2D Guideline).

Any suspected transmission via a medicinal product of an infectious agent is also considered a serious adverse reaction.

In this study, the safety events listed in Section 7.1 will be collected as study endpoints only and are not to be additionally recorded as an AE or SAE. These specific events are captured on the endpoint page of the eCRF only, and investigators must not report these on an AE/SAE page of the eCRF.

7.2.2 SAE reporting

To ensure patient safety, every SAE, regardless of causality, occurring after the patient's parent(s) or legal guardian(s) has provided informed consent and until 30 days after the last study visit or following the last administration of study treatment whichever is later must be reported to Novartis within 24 hours of learning of its occurrence. Any SAEs experienced after the 30 day period should only be reported to Novartis if the investigator suspects a causal relationship to study treatment.

[redacted]

All follow-up information for the SAE including information on complications, progression of the initial SAE and recurrent episodes must be reported as follow-up to the original episode within 24 hours of the investigator receiving the follow-up information. An SAE occurring at a different time interval or otherwise considered completely unrelated to a previously reported one must be reported separately as a new event.

Information about all SAEs is collected and recorded on the Serious Adverse Event Report Form; all applicable sections of the form must be completed in order to provide a clinically thorough report. The investigator must assess the relationship of each SAE to each specific component of study treatment (if study treatment consists of several components), complete the SAE Report Form in English, and submit the completed form within 24 hours to Novartis. Detailed instructions regarding the submission process and requirements for signature are to be found in the investigator folder provided to each site.

Follow-up information is submitted as instructed in the investigator folder. Each re-occurrence, complication, or progression of the original event must be reported as a follow-up to that event regardless of when it occurs. The follow-up information should describe whether the event has resolved or continues, if and how it was treated, whether the blind was broken or not and whether the patient continued or withdrew from study participation.

If the SAE is not previously documented in the Investigator's Brochure or Package Insert (new occurrence) and is thought to be related to the study treatment a Drug Safety and Epidemiology Department associate may urgently require further information from the investigator for health authority reporting. Novartis may need to issue an Investigator Notification (IN) to inform all investigators involved in any study with the same study treatment that this SAE has been reported. Suspected Unexpected Serious Adverse Reactions (SUSARs) will be collected and reported to the competent authorities and relevant ethics committees in accordance with EU Guidance 2011/C 172/01 or as per national regulatory requirements in participating countries.

In this study, safety events that are collected as study endpoints will not be recorded as SAEs and do not follow the above expedited reporting process (Section 7.2.1). These events will be monitored and reviewed regularly by Novartis or designee.

7.3 Reporting of study treatment errors

Medication errors are unintentional errors in the prescribing, dispensing, administration or monitoring of a medicine while under the control of a healthcare professional, patient/ parent(s)/ legal guardian(s) or consumer (EMA definition).

Study treatment errors and uses outside of what is foreseen in the protocol will be collected in the DAR (dose administration record) eCRF irrespective of whether or not associated with an AE/SAE and reported to Safety only if associated with an SAE.

Treatment error type	Document in Dose Administration (DAR) eCRF (Yes/No)	Document in AE eCRF	Complete SAE form
Unintentional study treatment error	Yes	Only if associated with an AE	Only if associated with an SAE

[redacted]

8 Data review and database management

8.1 Site monitoring

Before study initiation, at a site initiation visit or at an investigator's meeting, a Novartis representative will review the protocol and CRFs with the investigators and their staff. During the study, Novartis employs several methods of ensuring protocol and GCP compliance and the quality/integrity of the sites' data. The field monitor will visit the site to check the completeness of patient records, the accuracy of entries on the eCRFs, the adherence to the protocol and to Good Clinical Practice, the progress of enrollment, and to ensure that study treatment is being stored, dispensed, and accounted for according to specifications. Key study personnel must be available to assist the field monitor during these visits. Continuous remote monitoring of each site's data may be performed by a centralized Novartis CRA organization. Additionally, a central analytics organization may analyze data, identify risks and trends for site operational parameters, and provide reports to Novartis Clinical Teams to assist with trial oversight.

The investigator must maintain source documents for each patient in the study, consisting of case and visit notes (hospital or clinic medical records) containing demographic and medical information, laboratory data, electrocardiograms, and the results of any other tests or assessments. All information in the eCRFs must be traceable to these source documents in the patient's file. The investigator must also keep the original informed consent form signed by the patient's parent(s) or legal guardian(s) (a signed copy is given to the patient's parent(s) or legal guardian(s)).

The investigator must give the monitor access to all relevant source documents to confirm their consistency with the eCRF entries. Novartis' monitoring standards require full verification for the presence of informed consent, adherence to the inclusion/exclusion criteria, documentation of SAEs and of data that will be used for all primary variables. Additional checks of the consistency of the source data with the eCRFs are performed according to the study-specific monitoring plan. No information in source documents about the identity of the patients will be disclosed.

8.2 Data collection

Designated investigator staff will enter the data required by the protocol into the OC/RDC system. Designated investigator site staff will not be given access to the system until they have been trained.

Automatic validation procedures within the system check for data discrepancies during and after data entry and, by generating appropriate error messages, allow the data to be confirmed or corrected online by the designated investigator site staff. The Investigator must certify that the data entered into the electronic Case Report Forms are complete and accurate. After database lock, the investigator will receive copies of the patient data for archiving at the investigational site.

8.3 Database management and quality control

Novartis staff will review the data entered into the CRFs by investigational staff for completeness and accuracy and instruct the site personnel to make any required corrections or

[redacted]

additions. Queries are sent to the investigational site using an electronic data query. Designated investigator site staff is required to respond to the query and confirm or correct the data.

Concomitant medications entered into the database will be coded using the World Health Organization (WHO) Drug Reference List, which employs the Anatomical Therapeutic Chemical (ATC) classification system. Concomitant procedures, non-drug therapies and adverse events will be coded using the Medical Dictionary for Regulatory Activities (MedDRA) terminology.

Randomization codes and data about all study drug(s) dispensed to the patient will be tracked using an Interactive Response Technology (IRT) during Epoch 1. The system will be supplied by a vendor, who will also manage the database. The database will be sent electronically to Novartis.

The occurrence of relevant protocol deviations will be determined. After these actions have been completed and the database has been declared to be complete and accurate, it will be locked. Any changes to the database after that time can only be made after written agreement by Novartis Development management.

8.4 Data Monitoring Committee

An independent DMC (e.g. DMC involved in the core study CRFB002H2301) will be established to monitor the safety of the trial participants, to ensure that the trial is being conducted with the highest scientific and ethical standards, and make appropriate recommendations based on the data seen.

The DMC charter will include the DMC membership and responsibilities, the timing of DMC meetings, the content of the analysis report for the DMC meetings, and the communication with the Sponsor. The DMC will only make recommendations for changes in study conduct. The DMC will operate until the last patient has completed visit 301 (i.e. week 52 after the 1st study treatment in the core study) or discontinued the study whichever is the earliest.

8.5 Adjudication Committee

Not required.

9 Data analysis

The statistical analysis will be performed or directed by the Analytics Cluster of Novartis.

Three planned analyses will be performed in this study; two interim analyses (see Section 9.7) and one final analysis at the completion of the study. Additional interim analyses may be conducted on safety or efficacy data as required. Further technical details and discussions of the statistical considerations for each will be provided in the appropriate Statistical Analysis Plan (SAP), which will be finalized prior to first patient first visit (FPFV).

Descriptive statistics (mean, median, standard deviation, lower quartile (Q1), upper quartile (Q3), minimum, and maximum) will be presented for continuous variables. The number and percentage of patients in each category will be presented for categorical variables.

[redacted]

There are three treatment groups defined by the original treatment received at baseline in the core study:

- 0.2 mg Ranibizumab group
- 0.1 mg Ranibizumab group
- Laser treatment group

All results will be displayed by the above three treatment groups. If appropriate, within each of these three treatment groups, analyses may also be conducted separately for patients who received monotherapy or who switched treatment (by eye or by patient as applicable).

Two baselines are defined; the core baseline and the extension baseline. The core baseline will be the baseline of the core study. For statistical purposes, the last available non-missing values collected in study CRFB002H2301 or CRFB002H2301**E1** prior to or on visit 201 will be considered to be extension baseline values. Core baseline will be used as baseline for both efficacy and safety analyses. The extension baseline will be used for demographic summary of the extension study.

9.1 Analysis sets

The Extension Safety Set for this study is defined as the subset of the Safety Set of the study CRFB002H2301 who enters this study.

All analyses will be carried out on the Extension Safety Set and displayed by the original study treatment received at baseline in the core study unless otherwise specified.

9.2 Patient demographics and other baseline characteristics

Demographic information will be summarized for each treatment group and for all patients (total).

Relevant medical history and current medical conditions using the core baseline will be tabulated by system organ class (arranged alphabetically) and preferred term (arranged by decreasing frequency in the ranibizumab 0.2 mg treatment arm) according to the latest version of MedDRA. Separate tables will be presented for ocular and non-ocular histories and conditions.

The same approach will be used for such data collected after the last visit in the core study CRFB002H2301 and before the baseline visit in this extension study (Section 6.2).

ROP disease status will be defined as the value derived in the study CRFB002H2301.

9.3 Treatments (study drug, rescue medication, other concomitant therapies, compliance)

Exposure to investigational treatment will be presented by treatment group.

Patient disposition and reasons for discontinuation of study medication and discontinuation of study will be summarized by treatment group and total.

[redacted]

Concomitant therapies recorded during the core study (CRFB002H2301) and the extension study will be summarized by preferred term according to the latest version of the WHO Drug Reference List dictionary and ATC codes.

9.4 Analysis of the primary variable(s)

9.4.1 Variable(s)

The primary efficacy variable is the visual acuity (VA) in the better-seeing eye at the patient's fifth birthday as recorded by the investigator.

9.4.2 Statistical model, hypothesis, and method of analysis

If the primary hypothesis testing in the core study shows statistical significance, the primary objective of the extension study will be assessed by testing the superiority of ranibizumab 0.2 mg to laser as follows:

H_{01}: $\mu_{0.2\text{ mg Ranibizumab}} - \mu_{\text{Laser}} = 0$ versus H_{A1}: $\mu_{0.2\text{ mg Ranibizumab}} - \mu_{\text{Laser}} \neq 0$

where $\mu_{\text{Treatment Group}}$ is the unknown mean VA in the better-seeing eye at the fifth birthday of patients in the relevant treatment group.

The hypothesis will be tested by using stratified analysis of variance (ANOVA) using the Cochran-Mantel-Haenszel (CMH) test with the observed VA values as scores as the response variable and the treatment group as the factor (with levels 0.2 mg ranibizumab and laser). Stratification will be based on the ROP zone at the core study baseline. Superiority of ranibizumab 0.2 mg over laser will be concluded if the hypothesis H_{01} is rejected at the two-sided 5% significance level and $\mu_{\text{Ranibizumab 0.2 mg}} > \mu_{\text{Laser}}$.

If the primary hypothesis testing in the core study does not show statistical significance, only descriptive statistics (i.e., point estimate, and the 95% confidence interval) will be provided for the comparison between ranibizumab 0.2 mg and laser.

In addition, an unstratified two-way ANOVA using VA as the response variable and treatment group and zone as factors will be performed. Two-sided 95% confidence intervals, assuming normality, will be produced for the VA within each treatment arm and the difference in VA between a pair of treatment arms will be calculated from least squares means. No p-values will be displayed for this analysis.

Note that $\mu_{0.2\text{ mg Ranibizumab}}$ is estimated using those patients that received 0.2 mg ranibizumab at baseline in the core study CRFB002H2301. This includes patients that received laser treatment at a later date in either the core or extension study. Patients that permanently discontinue treatment with 0.2 mg ranibizumab are also included. However, patients for which the value of the primary variable is missing are excluded (see Section 9.4.3). A similar approach is taken for the 0.1 mg ranibizumab arm.

Similarly, μ_{Laser} is estimated using those patients that received laser at baseline in the core study CRFB002H2301. This includes patients that received 0.2 mg ranibizumab treatment at a later date in either the core or extension study. Patients that permanently discontinue treatment with laser are also included. However, patients for which the value of the primary variable is missing are excluded (see Section 9.4.3).

[redacted]

9.4.3 Handling of missing values/censoring/discontinuations

Missing data will not be imputed for the primary efficacy analysis. Hence, as stated in Section 9.4.2, the analysis of the primary variable is only conducted for patients with a non-missing value for VA.

9.4.4 Sensitivity analyses

The primary variable will also be assessed by using the randomized rather than the original treatment received in core study.

Additional analyses may be conducted for patients who switch treatment and patients who do not switch treatment.

Additional analyses may be conducted for patients who receive a medication which prohibits further use of ranibizumab or study laser. These analyses will be specified in the SAP.

9.5 Analysis of secondary variables

The analyses for objectives which are assessed up to a particular time (e.g. up to the patient's fifth birthday) will include data collected in both the core and extension studies.

No multiplicity adjustment will be used for the analysis of secondary variables.

No imputation of missing data will be used for the analysis of secondary variables unless specified in a scoring manual or similar.

Hence the analysis of all secondary variables will be based on the non-missing observations of patients that have not received a medication which prohibits further use of ranibizumab or study laser but may have permanently discontinued study treatment for another reason.

9.5.1 Efficacy variables

Continuous variables:

If the primary hypothesis testing of the core study shows statistical significance, the following objectives will be tested for the continuous variables below by using the same CMH test approach as the primary analysis.

The superiority of ranibizumab 0.1 mg to laser with regard to the VA of the better- seeing eye at the patient's fifth birthday

- The superiority of ranibizumab 0.2 mg to ranibizumab 0.1 mg with regard to the VA of the better-seeing eye at the patient's fifth birthday
- The three superiority tests comparing pairs of treatment arms will be repeated with regard to the VA of the worse-seeing eye at the patient's fifth birthday

Binary variables:

- The absence of all ocular structural abnormalities (see Section 3.5.2 for the definitions) at or before the patient's 2 years corrected age and fifth birthday.
- The recurrence of ROP up to 52 weeks post baseline visit in the core study
- The absence of active ROP at 52 weeks post baseline visit in the core study

[redacted]

The CMH test with ROP Zone as assessed at the baseline of the core study as stratification factor will be used for inference concerning binary secondary efficacy variables. However, the test will be based on proportions rather than a stratified ANOVA using the VA as a score. Mantel-Haenszel odds ratios and their two-sided 95% confidence intervals will be presented. Superiority will be defined as a rejection of the null hypothesis (equality of proportions) and the Mantel-Haenszel odds ratio is in favor of the hypothesized superior treatment.

Other efficacy variables:

Descriptive statistics will be provided for these efficacy variables by treatment arm:

- The absence of all ocular structural abnormalities (see Section 3.5.1 for the definitions) at or before 40 weeks post baseline visit in the core study
- The recurrence of ROP up to 40 weeks post baseline visit in the core study
- The absence of active ROP at 40 weeks post baseline visit in the core study
- The absence of each ocular structural abnormality considered individually (see Section 3.5.2 for the definitions) at or before the patient's 2 years' corrected age and at or before the patient's fifth birthday
- The number of ranibizumab injections received in the treatment of patients with ROP up to and including 40 weeks post baseline visit in the core study by eye and overall
- At the patient's 2 years corrected age and fifth birthday
    - The refraction in each eye: Refractive error will be categorized into myopia (spherical equivalent of 0.25 diopters or more of myopia) and high myopia (spherical equivalent of 5.00 diopters or more of myopia). The percentage of patients without myopia, with myopia, and with high myopia will be displayed.

9.5.2 Safety variables

Safety analyses will be assessed by using treatment-emergent AEs by treatment group and overall. Adverse events (overall AE, ocular AE, non-ocular AE) will be presented by primary system organ class (arranged alphabetically) and preferred term (arranged by decreasing frequency in the ranibizumab 0.2 mg treatment arm) using the most recent version of MedDRA available. Patients who experienced multiple AEs for a preferred term will be counted once, similarly for patients with multiple AEs per system organ class.

All other information collected (e.g., severity, relationship to study procedures and study treatment, leading to permanent study/study treatment discontinuation, and deaths) will be tabulated and listed as appropriate.

The number of hospitalizations/ prolongations of hospitalization due to an SAE will be summarized.

Deaths, SAEs, and AEs leading to permanent discontinuation of study treatment will be listed separately and, if appropriate, summarized by primary system organ class and preferred term.

Unless otherwise specified, AE data from the first study treatment in the core study will be included in all AE analyses. Vital signs, requirement for respiratory support and laboratory evaluations will be summarized by presenting descriptive statistics of raw values and change from baseline (if appropriate) by visit and treatment group.

[redacted]

Other safety data will be summarized and listed as appropriate.

[redacted]

9.7 Interim analyses

Two interim analyses will be performed by the Novartis CTT (see Section 3.5 for more details).

[redacted]

At the first interim analysis, descriptive statistics will be produced as appropriate and no inferential analyses will be conducted. The variables below will be explored.

- The absence of all ocular structural abnormalities as defined in Section 3.5.1.
- The recurrence of ROP up to 40 weeks post baseline visit in the core study
- The absence of active ROP at 40 weeks as defined in Section 3.5.1
- The number of ranibizumab injections received in the treatment of patients with ROP up to 40 weeks post baseline visit in the core study by eye and overall
- The type, frequency, and severity of ocular and non-ocular adverse events

At the second interim analysis, the variables below will be evaluated.

The appropriate CMH test (as specified in Section 9.5) will be used to analyze binary secondary efficacy variables at interim analysis 2. Suitable p-values and confidence intervals will be provided. No adjustment for multiplicity will be conducted. For other variables, descriptive statistics will be provided by treatment arm.

Binary variables:

- The absence of all ocular structural abnormalities (as defined in Section 3.5.2)

Other variables:

- The absence of each of the ocular structural abnormalities defined in Section 3.5.2
- The recurrence of ROP up to 52 weeks post baseline visit in the core study
- The absence of active ROP at 52 weeks post baseline visit in the core study as defined in Section 3.5.1
- The number of ranibizumab injections received in the treatment of patients with ROP up to 40 weeks post baseline visit in the core study by eye and overall

[redacted]

- Patient's physical development (standing/sitting height, leg length, weight and head circumference)
- The patient's health status (respiratory function, hearing function, duration of hospitalization from birth to first hospital discharge home, and weight at discharge)
- The type, frequency, and severity of ocular and non-ocular adverse events

[redacted]

- Visual acuity

Additional interim analyses will be conducted on safety or efficacy parameters as required.

[redacted]

9.8 Sample size calculation

The sample size calculation is not based on a power calculation. The number of patients enrolled in this study depends on the number of patients in the core study fulfilling the eligibility criteria of the extension study (see Section 4).

10 Ethical considerations

10.1 Regulatory and ethical compliance

This clinical study was designed and shall be implemented, executed and reported in accordance with the ICH Harmonized Tripartite Guidelines for Good Clinical Practice, with applicable local regulations (including European Directive 2001/20/EC, US CFR 21, and Japanese Ministry of Health, Labor, and Welfare), and with the ethical principles laid down in the Declaration of Helsinki.

10.2 Informed consent procedures

Eligible patients may only be included in the study after their parent(s) or legal guardian(s) have provided written (witnessed, where required by law or regulation), IRB/IEC-approved informed consent. Informed consent must be obtained before conducting any study-specific procedures (e.g. all of the procedures described in the protocol). The process of obtaining informed consent must be documented in the patient source documents.

Novartis will provide to investigators in a separate document a proposed informed consent form that complies with the ICH GCP guideline and regulatory requirements and is considered appropriate for this study. Any changes to the proposed consent form suggested by the investigator must be agreed to by Novartis before submission to the IRB/IEC, and a copy of the approved version must be provided to the Novartis monitor after IRB/IEC approval.

10.3 Responsibilities of the investigator and IRB/IEC

Before initiating a trial, the investigator/institution must obtain approval/favorable opinion from the Institutional Review Board/Independent Ethics Committee (IRB/IEC) for the trial protocol, written informed consent form, consent form updates, subject recruitment procedures (e.g., advertisements) and any other written information to be provided to the patient's parent(s) or legal guardian(s). Prior to study start, the investigator is required to sign a protocol signature page confirming his/her agreement to conduct the study in accordance with these documents and all of the instructions and procedures found in this protocol and to give access to all relevant data and records to Novartis monitors, auditors, Novartis Quality Assurance representatives, designated agents of Novartis, IRBs/IECs, and regulatory authorities as required. If an inspection of the clinical site is requested by a regulatory authority, the investigator must inform Novartis immediately that this request has been made.

10.4 Publication of study protocol and results

The key design elements of this protocol will be posted in a publicly accessible database such as clinicaltrials.gov. In addition, upon study completion and finalization of the study report the

[redacted]

results of this trial will be either submitted for publication and/or posted in a publicly accessible database of clinical trial results.

11 Protocol adherence

This protocol defines the study objectives, the study procedures and the data to be collected on study participants. Additional assessments required to ensure safety of patients/subjects should be administered as deemed necessary on a case by case basis. Under no circumstances is an investigator allowed to collect additional data or conduct any additional procedures for any research related purpose involving any investigational drugs under the protocol.

Investigators ascertain they will apply due diligence to avoid protocol deviations. If an investigator feels a protocol deviation would improve the conduct of the study this may be considered for a future protocol amendment, and unless such an amendment is agreed upon by Novartis and approved by the IRB/IEC and health authorities, where required, it cannot be implemented.

11.1 Protocol Amendments

Any change or addition to the protocol can only be made in a written protocol amendment that must be approved by Novartis, health authorities where required, and the IRB/IEC prior to implementation. Only amendments that are intended to eliminate an apparent immediate hazard to patients/subjects may be implemented immediately provided the health authorities are subsequently notified by protocol amendment and the reviewing IRB/IEC is notified. Notwithstanding the need for approval of formal protocol amendments, the investigator is expected to take any immediate action required for the safety of any patient included in this study, even if this action represents a deviation from the protocol. In such cases, the reporting requirements identified in Section 7 Safety Monitoring must be followed.

12 References

References are available upon request.

Adkins B, Leclerc C, Marshall-Clarke S [2004] Neonatal adaptive immunity comes of age. Nat Rev Immunol; 4(7):553-64.

Becker R, Hübsch S, Gräf MH, Kaufmann H [2002] Examination of young children with Lea symbols, Br J Ophthalmol, 86: 513-516

[redacted]

Boothe RG, Dobson V, Teller DY [1985] Postnatal development of vision in human and nonhumans primates. Ann Rev Neurosci; 8:495-545.

Chen YH, Chen S-N, Lien, R-I [2014] Refractive errors after the use of bevacizumab for the treatment of retinopathy of prematurity: 2-year outcomes. Retina 28: 1080-1087

[redacted]

Connolly BP, Ng EY, McNamara JA, et al [2002] A comparison of laser photocoagulation with cryotherapy for threshold retinopathy of prematurity at 10 years: Part 2. Refractive outcome. Ophthalmology; 109(5):936-41.

Cryotherapy for Retinopathy of Prematurity Cooperative Group [1988] Multicenter trial of cryotherapy for retinopathy of prematurity. Preliminary results. Arch Ophthalmol; 106(4):471-9.

Darlow BA, Ells AL, Gilbert CE, et al [2013] Are we there yet? Bevacizumab therapy for retinopathy of prematurity. Arch Dis Child Fetal Neonatal Ed; 98(2):F170-4.

De Pasquale MD, Pessolano R, Boldrini R, et al [2011] Continuing response to subsequent treatment lines with tyrosine kinase inhibitors in an adolescent with metastatic renal cell carcinoma. J Pediatr Hematol Oncol; 33(5):e176-9.

Edward DP, Kaufman LM [2003] Anatomy, development, and physiology of visual system. Pediatr. Clin North Am; 50(1):1-23.

Eremina V, Cui S, Gerber H, et al [2006] Vascular endothelial growth factor a signaling in the podocyte-endothelial compartment is required for mesangial cell migration and survival. J Am Soc Nephrol; 17(3):724-35.

Eremina V, Jefferson JA, Kowalewska J, et al [2008] VEGF inhibition and renal thrombotic microangiopathy. N Engl J Med; 358(11):1129-36.

European Medicines Agency (EMEA) [2006] Avastin: EPAR - scientific discussion. 24 Jan 2006. Available from:
http://www.ema.europa.eu/docs/en_GB/document_library/EPAR
_Scientific_Discussion/human/000582/WC500029262.pdf (Accessed 17 Sep 2014)

European Medicines Agency (EMEA) [2013] Eylea: EPAR - public assessment report. 21 Feb 2013. Available from:
http://www.ema.europa.eu/docs/en_GB/document_library/EPAR
_Public_assessment_report/human/002392/WC500135744.pdf (Accessed 17 Sep 2014)

Geloneck MM, Chuang AZ, Clark WL, et al [2014] Refractive outcomes following bevacizumab monotherapy compared with conventional laser treatment: a randomized clinical trial. JAMA Ophthalmol; Published online August 07, 2014 (doi:10.1001/jamaophthalmol.2014.2772)

Gerber HP, Hillan KJ, Ryan AM, et al [1999] VEGF is required for growth and survival in neonatal mice. Development; 126(6):1149-59.

Gilbert C [2008] Retinopathy of prematurity: a global perspective of the epidemics, population of babies at risk and implications for control. Early Hum Dev; 84(2):77-82.

Glade Bender JL, Adamson PC, Reid JM, et al [2008] Phase I trial and pharmacokinetic study of bevacizumab in pediatric patients with refractory solid tumors: a Children's Oncology Group Study. J Clin Oncol; 26(3):399-405.

Good WV, Early Treatment for Retinopathy of Prematurity Cooperative Group [2004] Final results of the Early Treatment for Retinopathy of Prematurity (ETROP) randomized trial. Trans Am Ophthalmol Soc; 102:233-48.

[redacted]

Hardy RJ, Good WV, Dobson V, et al [2004] Multicenter trial of early treatment for retinopathy of prematurity: study design. Control Clin Trials; 25(3):311-25.

Hartnett ME, Penn JS [2012] Mechanisms and management of retinopathy of prematurity. N Engl J Med; 367(26):2515-26.

Hoerster R, Muether P, Dahlke C, et al [2013] Serum concentrations of vascular endothelial growth factor in an infant treated with ranibizumab for retinopathy of prematurity. Acta Ophthalmol; 91(1):e74-5.

Honda S, Hirabayashi H, Tsukahara Y, et al [2008] Acute contraction of the proliferative membrane after an intravitreal injection of bevacizumab for advanced retinopathy of prematurity. Graefes Arch Clin Exp Ophthalmol; 246(7):1061-3.

Houston SK, Wykoff CC, Berrocal AM, et al [2013] Laser treatment for retinopathy of prematurity. Lasers Med Sci; 28(2):683-92.

Hu J, Blair MP, Shapiro MJ, et al [2012] Reactivation of retinopathy of prematurity after bevacizumab injection. Arch Ophthalmol; 130(8):1000-6.

International Committee for the Classification of Retinopathy of Prematurity [2005] The international classification of retinopathy of prematurity revisited. Arch Ophthalmol; 123(7):991-9.

Jang SY, Choi KS, Lee SJ [2010] Delayed-onset retinal detachment after an intravitreal injection of ranibizumab for zone 1 plus retinopathy of prematurity. J AAPOS; 14(5):457-9.

Jin K, Zhu Y, Sun Y, et al [2002] Vascular endothelial growth factor (VEGF) stimulates neurogenesis in vitro and in vivo. Proc Natl Acad Sci USA; 99(18):11946-50.

Kiely PM, Gillard-Crewther S, Nathan J, et al [1987] A comparison of ocular development of the Cynomolgus monkey and man. Clin Vision Sci; 1(3):269-80.

Kitamoto Y, Tokunaga H, Tomita K [1997] Vascular endothelial growth factor is an essential molecule for mouse kidney development: glomerulogenesis and nephrogenesis. J Clin Invest; 99(10):2351-7.

Kvarnström G, Jakobsson P [2005] Is vision screening in 3-year-old children feasible? Comparison between Lea Symbol chart and the HVOT (LM) chart Acta Ophthalmol. Scand. 83; 76-80

Law JC, Recchia FM, Morrison DG, et al [2010] Intravitreal bevacizumab as adjunctive treatment for retinopathy of prematurity. J AAPOS; 14(1):6-10.

Lee JY, Chae JB, Yang SJ, et al [2010] Effects of intravitreal bevacizumab and laser in retinopathy of prematurity therapy on the development of peripheral retinal vessels. Graefes Arch Clin Exp Ophthalmol; 248(9):1257-62.

Lee SJ, Kim SY, Yoo BC, et al [2011] Plasma level of vascular endothelial growth factor in retinopathy of prematurity after intravitreal injection of bevacizumab (E-abstract). Invest Ophthalmol Vis Sci; 52:3165A. Available from:
https://iovs.arvojournals.org/article.aspx?articleid=2360939

[redacted]

Malik AK, Beldwin ME, Peale F, et al [2006] Redundant roles of VEGF-B and PlGF during selective VEGF-A blockade in mice. Blood; 107(2):550-7.Micieli JA, Surkont M, Smith AF [2009] A systematic analysis of the off-label use of bevacizumab for severe retinopathy of prematurity. Am J Ophthalmol; 148(4):536-43.

Micieli JA, Surkont M, Smith AF [2009] A Systematic Analysis of the Off-Label Use of Bevacizumab for Severe Retinopathy of Prematurity. Am J Ophthalmol;148:536 –543

Mintz-Hittner HA, Kennedy KA, Chuang AZ, et al [2011] Efficacy of intravitreal bevacizumab for stage 3+ retinopathy of prematurity. N Engl J Med; 364(7):603-15.

Ng EY, Connolly BP, McNamara JA, et al [2002] A comparison of laser photocoagulation with cryotherapy for threshold retinopathy of prematurity at 10 years: Part 1. Visual function and structural outcome. Ophthalmology; 109(5):928-34.

Qiao-Grider Y, Hung LF, Kee CS, et al [2007] Normal ocular development in young rhesus monkeys (Macaca mulatta). Vision Res; 47(11):1424-44.

Royal College of Paediatrics and Child Health, Royal College of Ophthalmologists, British Association of Perinatal Medicine, BLISS [2008] Guideline for the Screening and Treatment of Retinopathy of Prematurity. UK Retinopathy of Prematurity Guideline. May 2008.

Ryan AM, Eppler DB, Hagler KE, et al [1999] Preclinical safety evaluation of rhuMAb VEGF, an antiangiogenic humanized monoclonal antibody. Toxicol Pathol; 27(1):78-86.

Şahin A, Şahin M, Cingü AK, et al [2013] Intravitreal bevacizumab monotherapy for retinopathy of prematurity. Pediatr Int; 55(5):599-603.

Sato T, Kusaka S, Shimojo H, et al [2009] Vitreous levels of erythropoietin and vascular endothelial growth factor in eyes with retinopathy of prematurity. Ophthalmology; 116(9):1599-603.

Sato T, Wada K, Arahori H, et al [2012] Serum concentrations of bevacizumab (Avastin) and vascular endothelial growth factor in infants with retinopathy of prematurity. Am J Ophthalmol; 153(2):327-33.

Smith AR, Hennessy JM, Kurth MA, et al [2008] Reversible skeletal changes after treatment with bevacizumab in a child with cutaneovisceral angiomatosis with thrombocytopenia syndrome. Pediatr Blood Cancer; 51(3):418-20.

Smith LE [2008] Through the eyes of a child: understanding retinopathy through ROP the Friedenwald lecture. Invest Ophthalmol Vis Sci; 49(12):5177-82.

Sonmez K, Drenser KA, Capone A Jr, et al [2008] Vitreous levels of stromal cell-derived factor 1 and vascular endothelial growth factor in patients with retinopathy of prematurity. Ophthalmology; 115(6):1065-70.

Wu WC, Kuo HK, Yeh PT, et al [2013] An updated study of the use of bevacizumab in the treatment of patients with prethreshold retinopathy of prematurity in Taiwan. Am J Ophthalmol; 155(1):150-8.

[redacted]

Wu WC, Yeh PT, Chen SN, et al [2011] Effects and complications of bevacizumab use in patients with retinopathy of prematurity: a multicenter study in Taiwan. Ophthalmology; 118(1):176-83.

Zepeda-Romero LC, Liera-Garcia JA, Gutierrez-Padilla JA, et al [2010] Paradoxical vascular-fibrotic reaction after intravitreal bevacizumab for retinopathy of prematurity. Eye (Lond); 24(5):931-3.

Zhou Y, Jiang Y, Bai Y, et al [2015] Vascular endothelial growth factor plasma levels before and after treatment of retinopathy of prematurity with ranibizumab. Graefes Arch Clin Exp Ophthalmol (DOI 10.1007/s00417-015-2996-0)

[redacted]
